# Phenotypic heterogeneity in the bacterial oxidative stress response is driven by cell-cell interactions

**DOI:** 10.1016/j.celrep.2023.112168

**Published:** 2023-02-26

**Authors:** Divya Choudhary, Valentine Lagage, Kevin R. Foster, Stephan Uphoff

**Affiliations:** 1 Department of Biochemistry, University of Oxford, Oxford, UK; 2 Department of Biology, University of Oxford, Oxford, UK

**Keywords:** cell-cell interactions, bacterial gene regulation, stress response, oxidative stress, phenotypic heterogeneity, single-cell imaging, machine learning

## Abstract

Genetically identical bacterial cells commonly display different phenotypes. This phenotypic heterogeneity is well known for stress responses, where it is often explained as bet hedging against unpredictable environmental threats. Here, we explore phenotypic heterogeneity in a major stress response of *Escherichia coli* and find it has a fundamentally different basis. We characterize the response of cells exposed to hydrogen peroxide (H_2_O_2_) stress in a microfluidic device under constant growth conditions. A machine-learning model reveals that phenotypic heterogeneity arises from a precise and rapid feedback between each cell and its immediate environment. Moreover, we find that the heterogeneity rests upon cell-cell interaction, whereby cells shield each other from H_2_O_2_ via their individual stress responses. Our work shows how phenotypic heterogeneity in bacterial stress responses can emerge from short-range cell-cell interactions and result in a collective phenotype that protects a large proportion of the population.

## Highlights

• Machine-learning model predicts single-cell heterogeneity
• Oxidative stress response heterogeneity is driven by cell-cell interactions
• Population protection increased by gradual adaptation of neighboring “barrier” cells
• Spatial H_2_O_2_ gradients give rise to cellular heterogeneity in mutagenesis

## Graphical abstract

**Figure:**
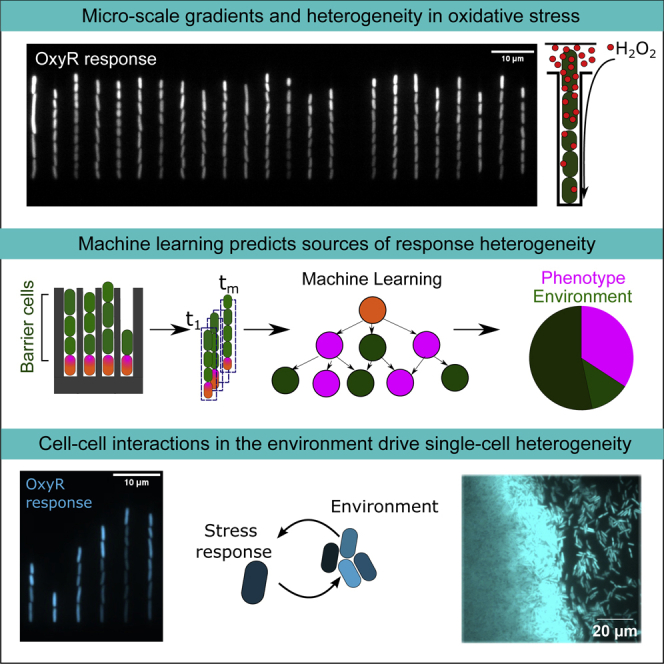

## Teaser

Clonal populations of bacteria often display heterogeneous phenotypes under stress. Here, using single-cell imaging and machine learning, Choudhary et al. show that heterogeneity in the response of *E. coli* cells to oxidative stress arises from short-range cell-cell interactions. This leads to a collective protection of the population.

## Introduction

Bacteria rely on sensory and gene regulatory systems to respond to changes in the conditions of their immediate environment. However, it has become clear that the stochastic nature of the underlying molecular processes can drive heterogeneity in the responses between individual cells. Consequently, even genetically identical cells that experience the same environmental conditions exhibit unpredictable variation in their phenotypes.^1^ Genes associated with stress responses appear to show particularly high levels of such variation,^2^ which may have evolved because it allows a genotype to survive.^3^^,^^4^^,^^5^^,^^6^ Such a bet-hedging strategy can increase the survival probability of the population, even though most cells do not maximize their individual fitness.^7^ Another explanation for the ubiquity of phenotypic heterogeneity during stress is that gene expression is inherently noisy.^2^^,^^8^^,^^9^^,^^10^^,^^11^ In bacteria, the number of molecules involved in sensing stress and regulating gene expression is often very small, such that stochastic fluctuations are unavoidable, and it may even be theoretically impossible for any stress response to be both fast and accurate at the same time.^12^ Hence, when the speed of gene induction is crucial for survival, noise in the response amplitude may be an inevitable by-product of the underlying molecular network.

Whether driven by bet-hedging or a speed-accuracy trade-off, heterogeneity in gene expression at a single-cell level is often attributed to the stochastic activation and binding of transcription factors,^13^ the synthesis of mRNAs and proteins in bursts,^11^ the random partitioning of molecules between sister cells during cell division,^14^ and changes in gene copy number over the course of DNA replication.^15^ These processes lead to variable abundances of proteins even when the average expression rates are constant across cells. Another potential source of variability is memory of past conditions.^6^^,^^16^^,^^17^ The duration of memory is generally set by the cell growth rate, which overall determines the balance between the production and dilution of molecules as cells elongate and divide.^18^^,^^19^ Because stress conditions affect growth rates, complex feedbacks can then arise between the stress level and gene expression dynamics. Variation in cell growth and morphology can also affect the influx, dilution, and reaction-diffusion dynamics of stressor molecules.^18^^,^^20^^,^^21^ Furthermore, cells in a population can interact and modulate their environment in response to stress.^22^^,^^23^^,^^24^ As such, it is notoriously difficult to disentangle the primary stochastic sources of phenotypic heterogeneity from secondary deterministic effects. This has been exemplified in studies that revisited the phage lysogenic switch and discovered that its apparent stochastic behavior can at least partly be explained by previously unobserved deterministic processes of the host cell.^25^^,^^26^^,^^27^

Here, we study the basis of cell-cell variability in the oxidative stress response of *E. coli* to hydrogen peroxide (H_2_O_2_). H_2_O_2_ is a major reactive oxygen species (ROS) that is generated as a by-product of aerobic metabolism under various stresses.^28^^,^^29^^,^^30^ In addition, bacteria can be exposed to H_2_O_2_ in the environment, for example, from host defenses or competing bacterial species.^31^^,^^32^^,^^33^ Reaction of H_2_O_2_ with iron leads to the formation of hydroxyl radicals that damage DNA and other essential biomolecules.^28^^,^^29^^,^^34^^,^^35^ The transcription factor OxyR senses an overabundance of H_2_O_2_ by oxidation and formation of a disulphide bond and subsequently induces the genes of H_2_O_2_ scavenging enzymes such as catalase and peroxidase.^29^^,^^36^^,^^37^ Oxidized OxyR also induces the glutaredoxin GrxA, which reduces OxyR and thereby enables deactivation of the response.^38^ H_2_O_2_ permeates the bacterial cell envelope,^39^ implying that a higher density of cells that actively scavenge H_2_O_2_ increases the survival of individual cells and, by extension, the stress tolerance at the population level.^40^^,^^41^^,^^42^

Exposure to ROS is rapidly lethal.^40^^,^^43^^,^^44^ Thus, genes of ROS scavenging enzymes are induced within minutes after H_2_O_2_ treatment.^34^^,^^36^ If there is a strong trade-off between response speed and accuracy, then it would be expected that these genes exhibit particularly noisy expression, and this was indeed observed in several studies with different types of ROS treatments.^34^^,^^45^^,^^46^^,^^47^ However, whether phenotypic variation during oxidative stress is truly the result of unavoidable molecular fluctuations, a population bet-hedging strategy, or caused by other factors is unknown. In addition to uncertainty about the functional consequences of phenotypic heterogeneity, its molecular origins are also complex and incompletely understood. To address this, we devised a strategy to understand the sources and consequences of oxidative stress response heterogeneity based upon analyzing the responses at the single-cell level as bacteria grow in a microfluidic device. Our work reveals that striking phenotypic heterogeneity in the oxidative stress response can emerge without the need for stochastic molecular noise but via cell-cell interactions.

## Results

### Strong cell-cell variability is observed in the H_2_O_2_ stress response

**Figure 1:**
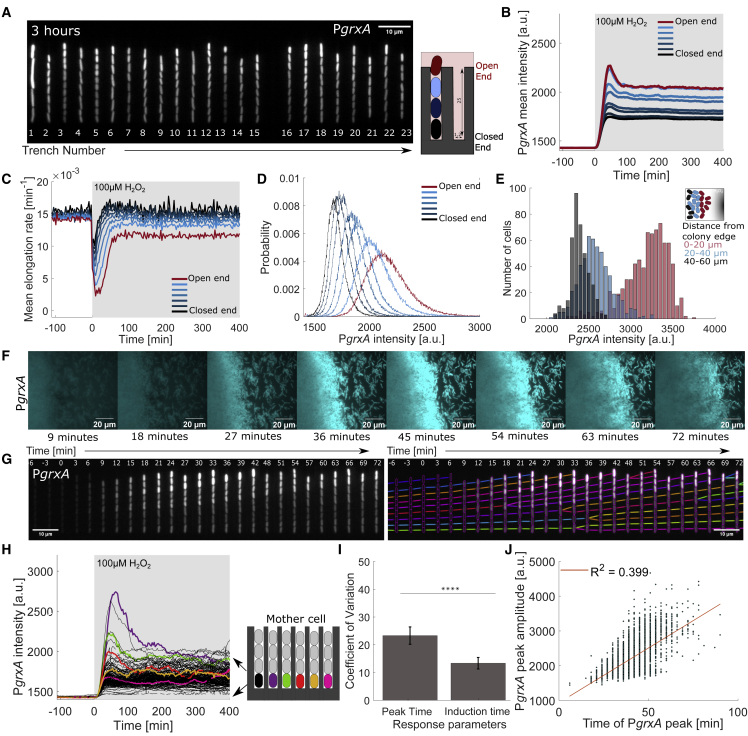
Spatial gradient in oxidative stress response (A) Snapshot of *E. coli* cells with transcriptional reporter P*grxA*-CFP in growth trenches after 3 h of 100 μM H_2_O_2_ treatment. (Video S1). (B) P*grxA*-CFP intensities with continuous 100 μM H_2_O_2_ treatment added at time 0 min (shaded area) averaged across cells at specific positions in the growth trench (black line: mother cells at closed end; red line: cells at open end, 3 experimental repeats). (C) Mean log elongation rate for cells at different positions in the growth trench with 100 μM H_2_O_2_ treatment added at 0 min (black line: mother cells at closed end; red line: cells at open end, 3 experimental repeats). (D) Distribution of P*grxA*-CFP intensities of cells with different number of barrier cells computed for 3 to 6 h after start of 100 μM H_2_O_2_ treatment (black line: mother cells at closed end; red line: cells at open end) (∼180,000 data points for steady state histograms, 3 experimental repeats). (E) Histograms of P*grxA*-CFP intensity for cells at different positions in a microcolony after 30 min of 10 mM H_2_O_2_ treatment. (F) P*grxA*-CFP snapshots of a microcolony under 10 mM H_2_O_2_ treatment (Video S7) (time post treatment annotated in the figures). (G) Kymograph of one growth trench with P*grxA*-CFP intensities and lineage tracing of cells over time with 100 μM H_2_O_2_ treatment added at time 0 min. (H) P*grxA*-CFP intensities of individual mother cells over time (example cells highlighted in color). (I) Temporal variation of the oxidative stress response across mother cells. Coefficient of variation (CV; SD/mean) of the response induction time after 100 μM H_2_O_2_ treatment (time until PgrxA-CFP > 1,480 a.u.) and the time to reach the P*grxA*-CFP peak intensity. CV was computed for all mother cells in a single experiment and collated for different repeats (bar chart represents the mean and SD, ^∗∗∗∗^p < 0.0001, 3 experimental repeats). (J) Correlation between P*grxA*-CFP peak amplitude and peak time (each dot represents one mother cell) (∼3,200 cells, 3 experimental repeats). Orange line represents the linear regression fit with R^2^ = 0.399.

We studied the oxidative stress response of cells in the “mother machine” microfluidic device for single-cell fluorescence imaging under constant growth conditions.^48^ The device consisted of an array of 25 μm long and 1.2 μm wide growth trenches that open to a perpendicular channel with constant medium flow (Figure 1A). Each trench contained 8 ± 2 cells with an average length of 2.6 ± 0.7 μm per cell (±SD) (Figure S1A). We used a strain with a transcriptional reporter for the OxyR response on a low copy-number plasmid (P*grxA*-CFP).^49^ A constitutively expressed P_RNAI_-mKate2 fluorescent protein served as a cytoplasmic label for automated cell segmentation and detection of cell division events. After a period of unperturbed growth, the growth medium flow was changed to medium containing 100 μM H_2_O_2_. Cells activated the OxyR response after a delay of 12.4 ± 2.1 min following the onset of treatment. P*grxA-*CFP expression peaked after 38 ± 10.8 min and subsequently stabilized at a lower steady-state expression level that was sustained by the constant flow of fresh medium with H_2_O_2_ into the growth trenches (Figure 1B; Video S1). Other transcriptional reporters regulated by OxyR, P*katG*-CFP and P*ahpC*-CFP, showed qualitatively similar expression dynamics (Figures S1B–S1D). As expected, the promoter activity (expression rate) of cytoplasmic label P_RNAI_-mKate2 stayed constant during treatment, but the overall intensity increased transiently (Figures S1E–S1G). This could be explained by a reduction in cell growth rate causing accumulation of cytoplasmic proteins. Indeed, treatment with 100 μM H_2_O_2_ caused an instantaneous drop in the rate of cell elongation (Figure 1C). However, following OxyR activation, most cells (91.86% ± 0.87%) recovered growth to pre-treatment rates, indicating complete adaptation to 100 μM H_2_O_2_, whereas cells with a *ΔoxyR* deletion are unable to induce the stress response and failed to recover (Figure S1H; Video S2). This assay, therefore, provides a way to investigate phenotypic heterogeneity under conditions where the stress response is both essential and effective. In addition, we later explore the impact of higher H_2_O_2_ concentrations on heterogeneity in cell fates (below).

Video S1. P*grxA*-SCFP3 fluorescence of wild-type (WT) cells with 100 μM H2O2 treatment from time 0, related to Figure 1

Video S2. Δ*oxyR* cells under 100 μM H2O2 treatment, related to Figure S1H Treatment was provided at time = 0. The RFP channel overlaid with YFP is shown in the movie to visualise cell boundary with the PRNAI-mKate2 expression and DNA mismatch detected as MutL-mYPet foci.

The magnitude of the oxidative stress response was highly variable across all cells present in the microfluidic trenches (Figure 1D). The mother machine device is popular for studies of cellular heterogeneity because the environmental conditions are believed to be so uniform that any phenotypic variation can be attributed to intracellular noise sources.^18^^,^^45^^,^^50^^,^^51^ However, this assumption is rarely tested rigorously. Here, we noticed that cells at the open end of the trenches exhibited higher OxyR reporter expression compared with cells located at the closed end during H_2_O_2_ treatment (Figures 1A, 1B, and 1D; Video S1). By analyzing cells according to their position in the trenches, we found that the average magnitude of the OxyR response decreased from the open to the closed end of the trench. The effect of H_2_O_2_ treatment on cell elongation was also reduced with increasing distance from the open end (Figure 1C). These observations suggested that the oxidative stress response is very sensitive to a cell’s local environment, wherein the stress level decays on a scale of a few micrometres away from the source of treatment. To test the relevance of this steep gradient in bacterial microcolonies, we applied 10 mM H_2_O_2_ treatment (higher concentration than microfluidics to account for cell density-dependent protection effect) onto a colony grown on agarose pads. This resulted in a higher P*grxA*-CFP response along the edge of the colony compared with the interior, which is consistent with the patterns in the mother machine (Figures 1E and 1F).

### A machine-learning model is able to predict single-cell responses to H_2_O_2_

Although a cell’s position in a trench accounted for a part of the observed phenotypic heterogeneity, we found that the magnitude of the stress response was variable even between individual cells located at the same position in the trenches, such as the “mother cells'' positioned at the closed ends (Figures 1G and 1H). Notably, the basal expression of P*grxA*-CFP without H_2_O_2_ treatment was uniform across cells, showing that the variation is indeed a consequence of environmental stress (Figure S1I). The magnitude of the initial P*grxA*-CFP response peak shortly after H_2_O_2_ treatment showed even higher cell-cell variation than the fluctuations of the response at steady state during prolonged treatment (Figure S1J). However, the activation time of the response after the start of treatment was much more uniform across mother cells, whereas the time to reach the expression peak was variable and correlated with the peak response magnitude of each cell (Pearson’s R = 0.399, p = 10^−15^) (Figures 1I and 1J). This indicates that while cells can reliably sense the onset of H_2_O_2_ stress and respond rapidly, the response magnitude depends on an unidentified factor that varies substantially between cells.

**Figure 2:**
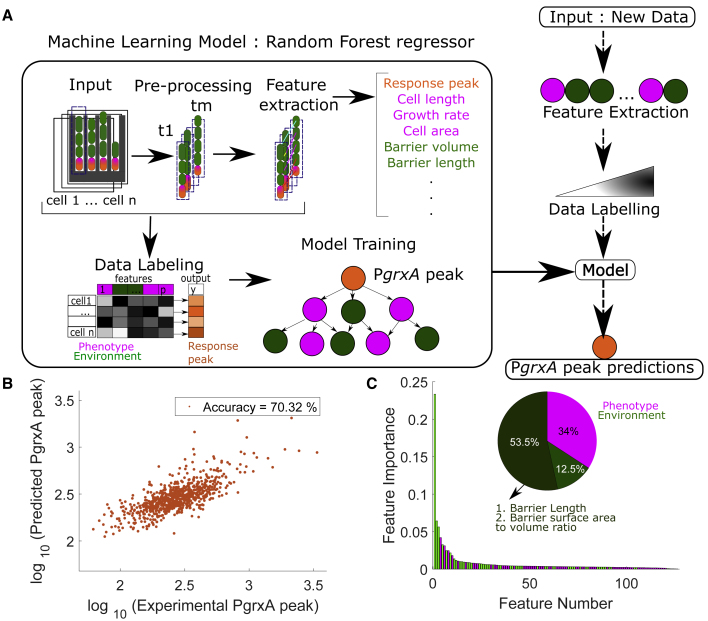
Machine learning predicts single-cell response heterogeneity (A) A random forest machine-learning model predicts P*grxA*-CFP peak intensities of 789 mother cells (orange). It uses features that describe the phenotypic characteristics of the mother cell (magenta) and the other cells in the local environment of each trench (barrier cells, green). (B) P*grxA*-CFP peak predicted by the model plotted against the experimentally measured P*grxA*-CFP peak (each dot represents one mother cell) (Data S1A). (C) Feature importance plots show the relative contribution of the 126 input features to the predictive power of the model (feature names in Data S1B). Barrier cell features (green) are more important than the mother cell features (pink). The two most important features are highlighted and account for 53.5% of the predictive power.

This heterogeneity could be caused by a variety of mechanisms, including molecular stochasticity in the specific regulatory circuits or general gene expression machinery,^11^^,^^13^ cell-to-cell variation in growth or morphology,^18^^,^^20^^,^^21^ variable cell-to-cell interactions,^22^^,^^23^^,^^24^ or differences in the local environment of cells.^24^^,^^52^ To pinpoint the mechanisms, we designed a machine-learning model using random forest regression (Figure 2A). We computed 126 features based on the time-series data of cell size, shape, and growth rates for each mother cell as well as the other cells present in the same trench (Data S1A). The model was trained to predict the peak intensity values of the P*grxA-*CFP reporter after the onset of H_2_O_2_ treatment from 80% of the observed mother cells and tested on the remaining 20% of mother cells (789, unseen during model training). The model predicted the magnitude of the oxidative stress response for individual cells with a mean accuracy of ∼70% (Figure 2B; Data S1B). Importantly, by its nature, the machine-learning model lacks the ability to predict cell-cell variability that arises stochastically. The power of the model, therefore, suggests that the primary cause of cell-cell variability is not driven by unpredictable molecular fluctuations. Rather, it has a deterministic origin. Further testament to the power of the model is that it was able to predict P*grxA*-CFP responses for cells treated with a range of H_2_O_2_ concentrations (37.5–100 μM) even though the concentration itself was unknown to the model. Hence, the features extracted from cell growth characteristics and morphology were sufficient to deduce the underlying H_2_O_2_ concentration (Figure S2A; Data S1C and S1D).

The machine-learning model also provides information on the sources of cellular heterogeneity. The features that contributed the most to the predictive power (∼66%) of the model related to the characteristics of what we term “barrier cells,” i.e., the cells located between the mother cell and the open end of each trench (Figure 2C). The two strongest features accounting for ∼53.5% of the total predictive power were (1) the cumulative length of the barrier cells in a trench and (2) the combined surface area-to-volume ratio of the barrier cells in a trench. In fact, a model that contained only features of the barrier cells and no features of the mother cell was still able to predict mother cell responses with high accuracy (Figure S2B; Data S1E and S1F). Hence, it appears that an individual cell’s stress response magnitude does not depend on its own characteristics or location but can be accurately predicted from the number and morphology of its neighbors. That is, the model suggests that the cell-cell variability rests primarily upon the effects of other cells on a focal cell. We next explored the mechanism by which the type and geometry of these intercellular interactions determine an individual cell’s stress response.

### The properties of neighboring cells drive oxidative stress response heterogeneity

**Figure 3:**
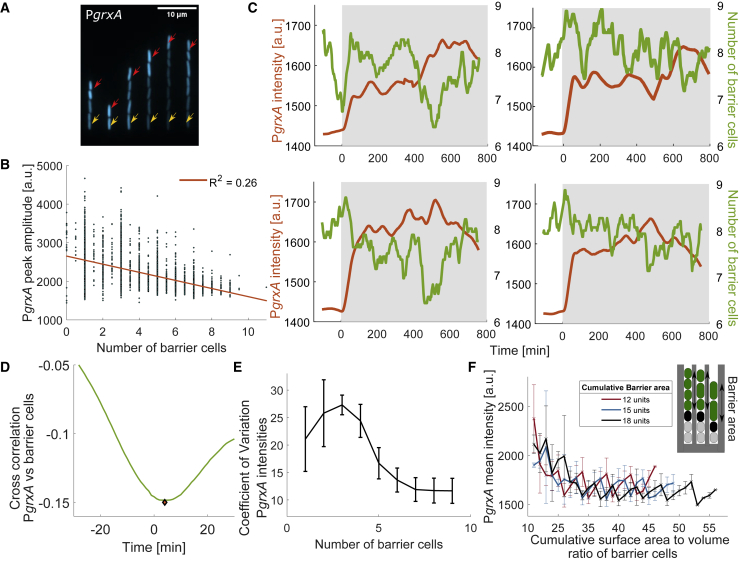
Variation in the spatial structure of the microenvironment explains response fluctuations (A) Snapshot of P*grxA*-CFP under 100 μM H_2_O_2_ treatment for growth trenches with variable number of cells (mother cells marked with yellow arrow, outermost cells with red arrow). (B) P*grxA*-CFP peak intensity amplitude under 100 μM H_2_O_2_ treatment plotted against the average number of barrier cells around the time of treatment (±9 min; each dot represents one mother cell) (∼3,250 cells, 3 experimental repeats). (C) Example time-traces of P*grxA*-CFP intensity (orange) for mother cells and the number of barrier cells in the same trench (green) with 100 μM H_2_O_2_ treatment added at 0 min (shaded area). Curves were smoothed using a moving mean filter with 45 min window. Note that barrier cell numbers fluctuate between 6 and 10 cells per trench during steady-state growth. (D) Mean temporal cross correlation for P*grxA*-CFP of mother cells against the number of barrier cells per trench (example time traces shown in C) when mean P*grxA*-CFP intensity has reached steady state from 2 h after start of 100 μM H_2_O_2_ treatment until end of experiment (∼11 h) (∼950 cells, 2 experimental repeats). Minima is represented by the orange diamond. (E) CV for P*grxA*-CFP intensity of mother cells with different number of barrier cells (CV: SD/mean was computed for all mother cells in a single experiment, error bars represent SD across experiments). (F) P*grxA*-CFP intensity for cells with the same total area of barrier cells (18, 15, or 12 units) plotted against the cumulative surface area-to-volume ratio of the barrier cells (3 experimental repeats).

To further test the apparent role of cell-cell interactions, we designed a microfluidic experiment with a variable number of cells per trench at the time of treatment (5 ± 2 cells) (Figure S3A) instead of filling all growth trenches completely. Under these conditions, the magnitude of the oxidative stress response of the mother cells became even more variable (Figures S3B and S3C). Notably, P*grxA*-CFP intensities of mother cells decreased when an increasing number of barrier cells were present, suggesting that these are protecting the mother cell from H_2_O_2_ (Figures 3A and 3B). The number of barrier cells fluctuates as cells divide and are pushed out of the open end of the trenches. Although the cell-average reporter expression was stable during constant H_2_O_2_ treatment, individual mother cells showed dynamic fluctuations at steady state (Figure 3C). Cross-correlation analysis showed that these expression fluctuations were negatively correlated with the variation in the number of barrier cells in the same trench (Figures 3D and S3D). The negative cross-correlation peaked at a lag time of 4.5 ± 1.5 min, showing that fluctuations in the number of barrier cells preceded changes in P*grxA*-CFP expression. These rapid changes in fluorescence signal are driven by dynamics in expression rate due to OxyR signaling as well as changes in cell growth rate that affect the rate of fluorescent protein dilution. Hence, the OxyR response is exquisitely sensitive and rapidly responds to very subtle changes in a cell’s microenvironment, such as the division or disappearance of a single cell in the vicinity. Based on calibration experiments with different H_2_O_2_ concentrations (Figures S3E and S3F), we inferred the local extracellular H_2_O_2_ concentration from the P*grxA*-CFP intensity of a cell. This showed an exponential decrease of H_2_O_2_ concentration from the open end of the trench, and each barrier cell decreased the local H_2_O_2_ concentration by 32.3% ± 4.6% (Figure S3G).

The response variation persisted even when restricting the analysis to mother cells with a fixed number of barrier cells, especially when that number was small (Figure 3E). Indeed, the machine-learning model pointed to the surface area-to-volume ratio of the barrier cells as an important feature that determines mother cell responses, in addition to the total volume of the barrier cells. To test this directly, we reasoned that an increased number of barrier cells at a constant total volume would result in a larger total surface area-to-volume ratio. As predicted, we found that the P*grxA*-CFP magnitude decreased with increasing surface area-to-volume ratio of barrier cells when their total volume was kept constant in the analysis (Figure 3F). Therefore, these measurements confirm the predictions of the machine-learning model that the number, size, and morphology of neighboring cells determine a cell’s dynamic response to H_2_O_2_.

### Intracellular scavenging enzymes create a local H_2_O_2_ gradient

**Figure 4:**
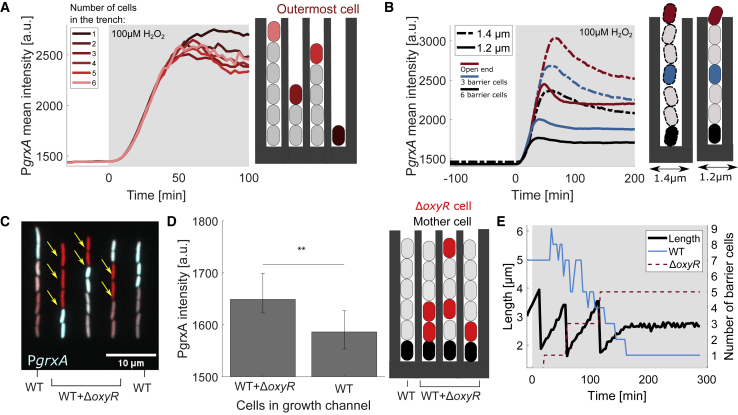
Intracellular scavenging enzymes create short-range local H_2_O_2_ gradients (A) Mean P*grxA*-CFP intensity for outermost cells in growth trenches (marked by red arrows in Figure 3A with total number of cells per trench ranging from 1 to 6). 100 μM H_2_O_2_ treatment added at 0 min (shaded area) (4 experimental repeats). Figure S3A shows the number of cells per curve. (B) P*grxA*-CFP mean intensities for trenches with widths of 1.2 (solid lines) and 1.4 μm (dashed lines) for cells with 0 (red), 3 (blue), and 6 (black) barrier cells (3 experimental repeats each). (C) Snapshot of merged P*grxA*-CFP (cyan) and mKate2 cell marker (red) intensity for trenches with a mix of wild-type (WT) and Δ*oxyR* strain under 100 μM H_2_O_2_ treatment (Video S3) (Δ*oxyR* cells marked with arrow, separate channel images in Figure S4B). (D) Mean P*grxA*-CFP intensity for WT mother cells in growth trenches containing a mix of barrier cells with at least one Δ*oxyR* cell or purely WT barrier cells (∼1,600 mother cells, 3 experimental repeats, error bars represent 25th and 75th percentiles, ^∗∗^p < 0.01). (E) Example time trace of length of a Δ*oxyR* cell situated at the closed end of a trench (black), and the number of WT (green) and Δ*oxyR* cells (red dashed) in the same trench. Number of WT cells decreases as the Δ*oxyR* cells grow in the trenches and push them out (Videos S4 and S5), demonstrating that a Δ*oxyR* cell can only grow when protected by WT cells.

We next sought to understand the mechanistic basis for the cell-cell interactions underlying phenotypic heterogeneity. We again used microfluidic chips in which the trenches were only partially filled with cells. The outermost cells in each growth trench exhibited the same level of stress response irrespective of their distance from the open end, showing that the geometry of the trenches itself does not restrict H_2_O_2_ diffusion (Figures 3A and 4A). Importantly, this demonstrates that the microfluidic device itself does not create gradients in the stressor that could explain cell-cell differences in the responses. If the cells themselves are indeed generating the observed patterns, this suggests that changes in cell density will impact the cell-cell variability. To test the effects of cell density, we next designed a variation on our mother machine device that had wider trenches, increasing them from 1.2 to 1.4 μm. As again predicted by the importance of cell-cell effects, this local reduction in cell density reduced both the barrier effect (Figure 4B) and cell-cell variation (Figure S3H). Hence, a decrease in cell density permits a higher and more uniform H_2_O_2_ flux. However, this effect could be caused by the cell mass passively blocking H_2_O_2_ diffusion or by active degradation of H_2_O_2_ by the barrier cells.

We hypothesized that a role for active degradation was likely because H_2_O_2_ is rapidly scavenged by catalase, which is induced by the OxyR response. The Damköhler number (a dimensionless quantity that relates the reaction rate to the rate of diffusion) is ∼4·10^3^ for the ratio of the catalase scavenging rate (5.4·10^4^ s^−1^)^37^ to the diffusion of H_2_O_2_ across the *E. coli* cell envelope (1.6·10^−5^ m/s).^39^ Hence, the scavenging rate is limited by the diffusion into cells, which is consistent with our finding that the protective effect of the barrier cells was determined to a large extent by their surface area. To experimentally test for the importance of H_2_O_2_ scavenging in creating the observed exponential stress gradient along the trenches in the device, we loaded trenches with a mix of a Δ*oxyR* strain and a wild-type strain with P*grxA*-CFP reporter (Figure 4C). Δ*oxyR* cells cannot induce catalase and other oxidative stress response genes, which limits their ability to degrade high amounts of H_2_O_2_ coming in from the environment.

As expected, if H_2_O_2_ scavenging is important, we observed that the gradient in P*grxA*-CFP reporter intensity was disrupted by the presence of Δ*oxyR* cells (Figure 4C). Moreover, wild-type cells positioned behind Δ*oxyR* cells showed elevated stress responses (Figures 4D and S4A–S4C; Video S3). Δ*oxyR* barrier cells caused no reduction in local H_2_O_2_ concentration (Figures S4A–S4C) unlike the ∼30% reduction by wild-type barrier cells (Figure S3G). In fact, wild-type cells had a marked protective effect on Δ*oxyR* cells, consistent with bulk culture measurements.^40^ On their own, Δ*oxyR* cells were unable to grow during 100 μM H_2_O_2_ treatment (Figure S1H; Video S2) but with wild-type cells acting as a barrier, Δ*oxyR* cells were able to grow until the wild-type cells exited the trench (Figure 4E; Videos S4 and S5). We also studied the effect of Δ*oxyR* cells in a bacterial colony by again mixing them with wild-type strain carrying the P*grxA*-CFP reporter. Here, we observed a stronger stress response than in a clonal wild-type colony and no longer saw a steep spatial gradient from the edge to the interior (Figures S4D–S4F). This is again consistent with the importance of H_2_O_2_ scavenging rather than cells passively blocking H_2_O_2_. To explore if cell mass had any protective effect, we loaded trenches with a mixture of wild-type cells and inert cells that had been chemically fixed. Like Δ*oxyR* cells, inert cells caused no reduction in the stress level of neighboring cells (Figures S4G and S4H).

Video S3. Mix of WT and Δ*oxyR* cells under 100 μM H2O2 treatment, related to Figures 4C and 4D Treatment was provided at time = 0. The RFP channel overlaid with CFP and YFP is shown in the movie to visualise cell boundary with PRNAI-mKate2 expression, P*grxA*-SCFP3 expression and DNA mismatch detected as MutL-mYPet foci.

Video S4. The presence of WT cells acting as a barrier rescues the growth of Δ*oxyR* cells until the WT cells exited the trench, related to Figure 4E Mix of WT and Δ*oxyR* cells in growth trenches 1, 2, 4 and 7 cells with Δ*oxyR* cells occupying mother cell position in trenches 1, 2 and 4. Treatment was provided at time = 0. The RFP channel overlaid with CFP and YFP is shown in the movie to visualise cell boundary with PRNAI-mKate2 expression, P*grxA*-SCFP3 expression and DNA mismatch detected as MutL-mYPet foci.

Video S5. The presence of WT cells acting as a barrier rescues the growth of Δ*oxyR* cells until the WT cells exited the trench, related to Figure 4E Mix of WT and Δ*oxyR* cells in the right growth trench with Δ*oxyR* cell occupying the mother cell position. Treatment was provided at time = 0. The RFP channel overlaid with CFP and YFP is shown in the movie to visualise cell boundary with PRNAI-mKate2 expression, P*grxA*-SCFP3 expression and DNA mismatch detected as MutL-mYPet foci.

### Barrier cells obtain high H_2_O_2_ tolerance via gradual adaptation

We next sought to understand how the cells at the top of the trench are able to survive and provide protection for other cells in the face of high concentrations of H_2_O_2_. Elongation rates of the outermost cells in the trenches show that >50 μM H_2_O_2_ will lead to growth inhibition in the absence of cellular cross-protection (Figure S5A). Consistent with this, the onset of our standard 100 μM H_2_O_2_ treatment completely halted the growth and division of ∼1–2 outermost barrier cells per trench. However, the cells in the interior of the trenches only transiently slowed in growth and quickly recovered unperturbed elongation and division rates despite ongoing H_2_O_2_ treatment (Figure 1C). These surviving interior cells divide and push live progeny toward the stress source and thereby replace the barrier cells that had been stalled in growth by the sudden onset of treatment. This suggests that the barrier cells that persist are those that are given time to adapt to a gradual increase in H_2_O_2_ concentration. Such gradual adaptation is known to enhance survival of higher stress levels.^34^^,^^53^^,^^54^

**Figure 5:**
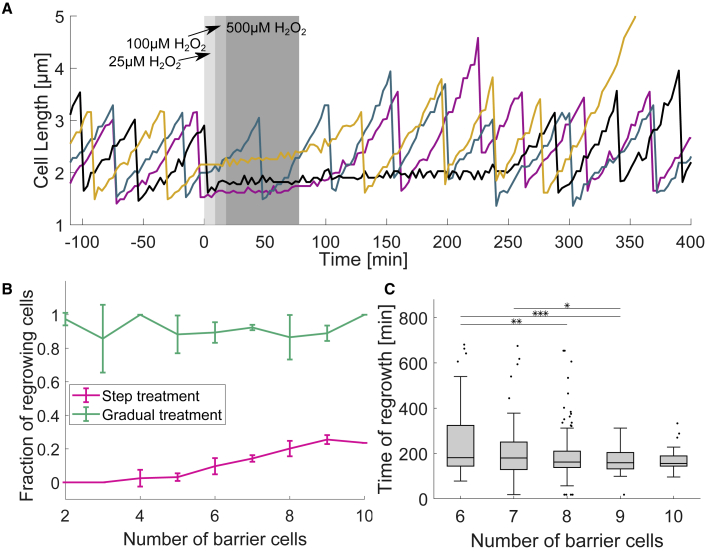
Local environmental variation determines divergent cell fates after H_2_O_2_ treatment (A) Individual mother cell length traces with gradual increase in H_2_O_2_ concentration from 25 to 100 μM to 500 μM (shaded area), followed by recovery without treatment. (B) Proportion of mother cells that recover growth within ∼11 h after the removal of 500 μM H_2_O_2_ treatment that was applied in a single step (pink) or gradually (green, as illustrated in A) plotted against number of barrier cells at the time of treatment (Video S6) (≥3 experimental repeats each; line plot with mean and standard deviation). (C) Time of regrowth of mother cells after step 500 μM H_2_O_2_ treatment plotted against number of barrier cells at the time of treatment. (∼300 cells; boxplot with median, 25th and 75th percentiles; ^∗^p < 0.05, ^∗∗^p < 0.01, ^∗∗∗^p < 0.001).

To test if this “priming” effect underlies the high stress tolerance of the barrier cells, we compared the impact of the sudden onset of high levels of H_2_O_2_ with a gradual onset (Figure 5A). A sudden 1 h pulse with 500 μM H_2_O_2_ concentration stalled the growth of 99% of mother cells (Figure 5B), and only 13% of mother cells subsequently recovered growth (Figure S5B; Video S6). Consistent with our observations of cross-protection at 100 μM H_2_O_2,_ these individuals were protected by more barrier cells than those that irreversibly stopped growth (Figure 5B). Furthermore, the delay before the surviving cells started to regrow after treatment removal was variable and negatively correlated with the number of barrier cells (Figures 5A and 5C). When a gradual increase of H_2_O_2_ concentration was applied, again reaching up to 500 μM in 18 min and then kept constant for an hour, many more of the cells survived (Figure 5B). The result is consistent with the importance of priming for generating a subpopulation of stress-tolerant barrier cells. However, the actual adaptation process of the barrier cells during continuous treatment is more complex because it involves both a temporal and a spatial gradient in stress level.

Video S6. Cell death under 500 μM H2O2 treatment, related to Figure S5B RFP channel fluorescence of WT cells with 500 μM H2O2 treatment from time 0 min to 60 min. Propidium iodide was added ∼11.5 h post treatment removal.

**Figure 6:**
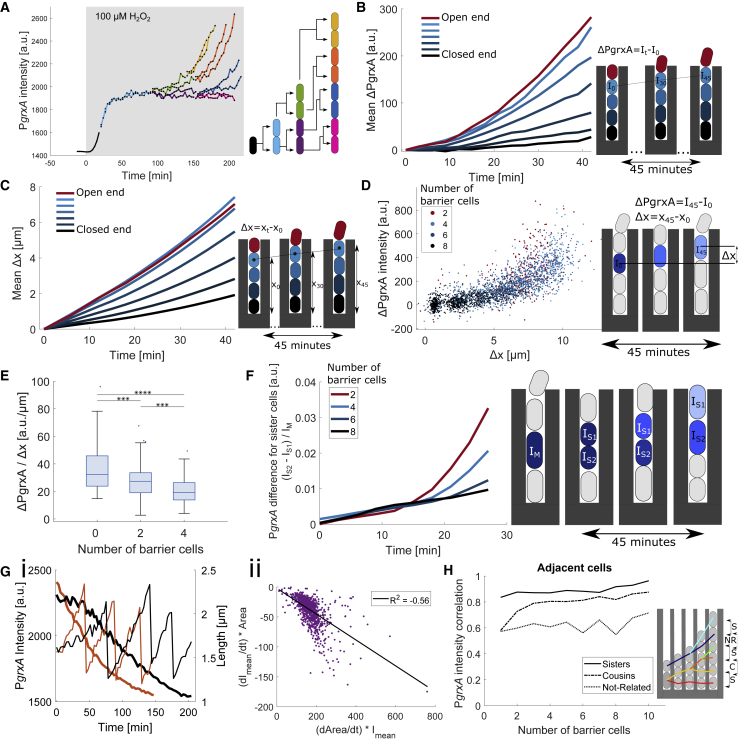
Spatiotemporal response dynamics overwrite cell lineage-associated memory (A) P*grxA*-CFP intensities for a representative mother cell and its progeny that move toward the opening of the growth trench. 100 μM H_2_O_2_ treatment added at 0 min (shaded area). (B) Effect of cell movement on P*grxA*-CFP expression in a spatial H_2_O_2_ gradient: mean increase in P*grxA*-CFP intensity (ΔPgrxA) over time when the population response has reached steady state (from 2 h after start of 100 μM H_2_O_2_ treatment until end of experiment, ∼11 h). Each curve represents cells moving toward the trench opening from a different starting position (black line: mother cells at closed end; red line: cells at open end) (3 experimental repeats). (C) Effect of cell position in the growth trench on the movement speed: each curve shows the mean movement Δx toward the trench opening of cells with different starting position (black line: mother cells at closed end; red line: cells at open end) (3 experimental repeats). (D) Response induction rate depends on the cell movement speed: each point represents the total increase in P*grxA*-CFP and total movement of a single cell traced for 45 min. Colors represent the starting position according to the number of barrier cells (∼2,800 total number of cells, 3 experimental repeats). (E) Response induction rate depends on cell position in the growth trench: relative increase in P*grxA*-CFP per distance for cells that move 7 ± 0.2 μm in 45 min. Number of barrier cells represents the starting position (∼200 total number of cells, 3 experimental repeat, ^∗∗∗^p < 0.001, ^∗∗∗∗^p < 0.0001). (F) Responses of sister cells diverge over time: P*grxA*-CFP intensity difference between sister cells (S1 and S2) followed for 30 min post division when the response has reached steady state. Intensity differences were normalized by the intensity of the cell they arose from. Number of barrier cells represents the starting position at the time of cell division (∼5,400 pairs, 3 experimental repeats). (G) Heterogeneous growth rates explain variability in the deactivation rate of the response: (i) P*grxA*-CFP intensity (thick lines) and cell lengths (thin lines) of 2 representative mother cells (black and orange plots respectively) after removal of 500 μM H_2_O_2_ at time 0 min, showing that faster growth leads to a faster decay. (ii) Correlation between P*grxA*-CFP intensity decay (dI_mean_/dt) and cell growth rate (dArea/dt) after removal of 500 μM H_2_O_2_ (∼1,000 mother cells). (H) Effect of cell position on response memory: Pearson’s correlation coefficient for P*grxA*-CFP intensities of adjacent cell pairs with variable number of barrier cells, related as sisters (Ss; solid), cousins (Cs; dash-dotted), or not related (NR; dotted) (3 experimental repeats).

To understand the spatiotemporal dynamics of adaptation, we tracked individual cells as they moved from birth at the closed end of the trench until they exited the open end (Figure 6A). P*gxrA*-CFP expression increased rapidly over time as cells traverse the spatial H_2_O_2_ gradient (Figures 6B and S5C). This observation can be explained by two effects: first, the exponential increase in H_2_O_2_ concentration along the length of the trench, and second, the increasing speed of movement as cells are pushed by exponentially growing cells located deeper in the trench (Figure 6C). The speed of movement varies between cells due to fluctuations in elongation rates and the number of cells per trench. Cells that had the same number of barrier cells and moved faster to the trench opening showed a steeper response than slower-moving cells (Figure 6D). When mobile cells experience a gradient of H_2_O_2_ in space and time, successful adaptation requires that the induction rate of scavenging enzymes matches the increasing influx of H_2_O_2_. Indeed, movement speed and the response induction rate were correlated in individual cells (Pearson correlation coefficient = 0.76) (Figure 6D). Next, in order to isolate the effect of the spatial gradient on the response induction rate, we analyzed cells that moved with similar speed. In this case, cells with fewer barrier cells had a higher response induction rate because they experience a steeper spatial H_2_O_2_ gradient (Figure 6E). Together, the multiplicative effects of accelerated movement and increasing stress gradient lead to a rapid induction of the response as cells are pushed toward the trench opening.

### A rapid response to the environment overwrites cellular memory

In principle, a cell’s gene expression level is shaped by its immediate response to the current local environment as well as its memory of past stress exposure.^16^ We thus explored if any of the response heterogeneity that we observe can be traced back to a cell’s lineage history (Figure 6F). When H_2_O_2_ treatment was stopped, reporter intensities started to drop quickly and decayed exponentially with a half-life that matched the cell doubling time, confirming that CFP is a stable protein and diluted by cell division (Figures S5D and S5E). The P*grxA*-CFP decay rate was variable across cells but closely correlated with the growth rate, which explained the observed cell-cell variation in the response deactivation (Figure 6G). Cytoplasmic proteins are randomly equipartitioned at cell division, so the CFP intensities of sister cells were closely correlated immediately after birth (Figure S6A). However, during constant H_2_O_2_ treatment, the intensity of the cell located closer to the open end of the trench quickly diverged from the sister (Figures 6F and S6B). Specifically, the intensity difference between the two sisters increased exponentially over time because the cell closer to the open end of the trench experiences a higher H_2_O_2_ concentration and itself acts as a barrier for the cell located below. This effect was much less pronounced for sisters born with a larger number of barrier cells because they move more slowly in a shallower H_2_O_2_ gradient. These patterns suggest that any stress response memory is erased quickly for cells that move rapidly up the H_2_O_2_ gradient at the open end of the trenches. However, cells that experience more uniform conditions could, in principle, maintain memory across generations.

To test this, we quantified the difference in CFP reporter intensities between adjacent cells in the same trench that were either related as sisters (separated 1 generation before), cousins (2 generations separated), or not related (>2 generations separated). Sister pairs were more correlated than cousins, and cousins were more correlated than distantly related cell pairs (Figures 6H and S6C), confirming the presence of cross-generational response memory. However, the correlation between cousins dropped to the level of unrelated cells when the number of barrier cells was small (Figures 6H and S6D). We further tested cell memory using repeated short pulses of H_2_O_2_ treatment to understand whether a past response affected a subsequent response. We saw a low level (∼25%) of correlation in the response intensities between subsequent pulses of the same mother cell (Figures S6E–S6G). Therefore, oxidative stress response memory can persist over generations when the environment is constant, but changing environmental conditions dictate the instantaneous response level and effectively overwrite cell lineage memory.

### Spatial gradients in H_2_O_2_ lead to cellular heterogeneity in mutagenesis

**Figure 7:**
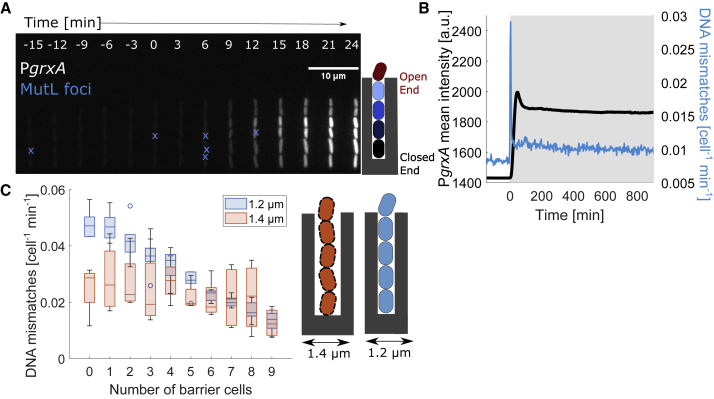
Spatial heterogeneity in H_2_O_2_ concentration causes cell-to-cell variation in mutagenesis (A) Kymograph of one growth trench with P*grxA*-CFP intensities under 100 μM H_2_O_2_ treatment added at time 0 min. Blue crosses represent MutL-mYPet mismatch foci. (B) Mean rate of DNA mismatch foci (per cell per minute, blue) and P*grxA*-CFP mean intensity (black) for all cells in the growth trenches (3 experimental repeats). (C) Dependence of the mismatch rate on cell position: amplitude of the DNA mismatch rate peak for cells with different numbers of barrier cells under 100 μM H_2_O_2_ treatment for growth in 1.2 (blue) and 1.4 μm (orange) wide trenches (Video S7) (3 experimental repeats each).

It has been shown that variation in the timing^51^^,^^55^ or magnitude of a stress response^56^ can cause cell-to-cell variation in mutation rates. We were therefore interested in whether the phenotypic heterogeneity driven by cell-cell interaction in our experiments could also lead to mutational heterogeneity. We previously reported that H_2_O_2_ treatment causes a burst of mutations prior to induction of the oxidative stress response in *E. coli*.^34^ Here, we used the same approach with a MutL-mYPet fusion (which forms foci at sites of DNA mismatches) to monitor the occurrence of DNA replication errors in single cells.^57^^,^^58^ Cells showed a burst of DNA replication errors immediately after the onset of treatment (Figures 7A and 7B), and the magnitude of this burst decreased steeply with an increasing number of barrier cells (Figure 7C). Moreover, the spatial gradient in mutagenesis was lost when we grew cells in wider trenches (Figure 7C), consistent with the uniform stress response observed under these conditions. Finally, bacterial colonies with 10 mM H_2_O_2_ treatment exhibited a higher frequency of DNA mismatch foci along the edge of the colony compared with the interior (Video S7). Therefore, mutational heterogeneity can arise when local cellular interactions generate cell-cell variability in stress response expression.

Video S7. WT colony under 10 mM H2O2 treatment, related to Figures 1F and 7 Images were taken every 3 min, starting 6 min post treatment. The YFP channel showing MutL-mYPet foci overlaid with P*grxA*-SCFP3 is shown.

## Discussion

Phenotypic heterogeneity within bacterial populations has been well documented, but its origins are difficult to pinpoint, and its purpose is debated. This cell-cell variability is often attributed to bet hedging but could also arise due to unavoidable biochemical noise or an underlying deterministic process that appears random because it has not been thoroughly characterized. These scenarios are not necessarily mutually exclusive, and a part of the apparent heterogeneity may also stem from non-biological measurement noise. Studies of the oxidative stress response in bacteria have revealed characteristics that partially support each of these models.^18^^,^^41^^,^^45^^,^^47^^,^^59^ To help resolve this uncertainty, here we have characterized the spatiotemporal behavior of single *E. coli* cells growing in a defined environment under a constant treatment of H_2_O_2_. In contrast to the expectation from a stochastic process, we found that individual cells modulate their gene expression extremely precisely to the local H_2_O_2_ concentration. Moreover, the induction of scavenging enzymes generates steep H_2_O_2_ gradients, with each cell contributing to a ∼30% reduction of H_2_O_2_ in its vicinity. The result is that the response of a focal cell is defined by the properties of its neighbors, which generates striking phenotypic heterogeneity via cell-cell interactions.

Our work does not exclude the possibility that stochastic processes become important for phenotypic heterogeneity in the oxidative stress response under lethal conditions, where the heterogeneity in fate of the cells can be a result of stochastic biological events like DNA damage not explored in detail in this study. Nevertheless, our findings make clear that striking cell-cell variability can emerge without the need for such stochastic underpinnings. The accuracy of the response to the local environment is likely based on the rapid uptake of H_2_O_2_ together with its high reactivity and specificity toward OxyR.^60^ Induction of scavenging enzymes and GrxA reductase lowers the intracellular H_2_O_2_ concentration and deactivates OxyR in a negative feedback loop, which increases response speed and accuracy in general.^61^ Because oxidative stress is mutagenic, the emergent gradients in H_2_O_2_ concentration also caused spatial variation in the frequency of DNA replication errors across cells. This process can thereby diversify mutation rates among cells growing in a structured population. Indeed, our observation that H_2_O_2_-induced mutation rates decrease with increasing cell density matches results obtained in liquid bulk culture.^62^^,^^63^

We find that individual cells can rapidly modulate their oxidative stress response according to the ROS levels in their immediate environment, which improves their chances of survival (Figures 4, 5, and S5). However, this individual response can also result in the emergence of a collective behavior whereby cells that are closest to a stressor protect those that are further away. While sudden exposure to high H_2_O_2_ concentrations can kill cells on the outside of a dense population, the initial H_2_O_2_ gradient created by these cells allows the survivors in the interior to regrow and reestablish the protective gradient. This collective response, therefore, also has temporal component by allowing a subpopulation of cells to experience a gradual increase in stress and obtain high tolerance via priming. This all suggests that there can be both an individual and group benefit to the generation of this phenotypic heterogeneity.

### Limitations of the study

A limitation we encountered in our study is the difficulty to account for the effects of cell death during H_2_O_2_ treatment. The fluorescence intensity of a transcriptional reporter (e.g., P*grxA*-CFP) provides a readout for the H_2_O_2_ level in live cells only. Our time-lapse measurements can distinguish live and dead cells based on the growth rate. We confirmed using propidium iodide staining that mother cells that stopped growing for multiple hours during H_2_O_2_ treatment are indeed dead (Figure S5B). However, it was not possible to reliably distinguish live and dead barrier cells from the growth rate because the observation time is limited as these cells are quickly pushed out of the trenches. Hence, we were unable to characterize quantitatively how the death of barrier cells affects the H_2_O_2_ gradient. Other indicators for cell viability and/or reporters for the H_2_O_2_ level in cells could resolve this issue in future studies. Another limitation related to the use of a transcriptional CFP reporter protein is that it can only indirectly inform about response memory. Further insight could be gained by measuring the levels of the native stress response proteins using translational fluorescent fusions or biochemical quantification techniques.

## STAR★Methods

### Key resources table

**Table:** 

REAGENT or RESOURCE	SOURCE	IDENTIFIER
**Bacterial and virus strains**		

AB1157, Δ*flhD*, P_RNAI_-mKate2, mutL-mYpet (SU178)	Lagage et al.^34^	N/A
AB1157, Δ*flhD*, P_RNAI_-mKate2, mutL-mYPet, carrying pUA139 P*grxA*-SCFP3A Kan (SU777)	Lagage et al.^34^	N/A
AB1157, Δ*flhD*, P_RNAI_-mKate2, mutL-mYpet, Δ*oxyR*, carrying pUA139 P*grxA*-SCFP3A kan (PSU044) (SU802)	Lagage et al.^34^	N/A
AB1157, Δ*flhD*, P_RNAI_-mKate2, mutL-mYpet, carrying pUA066-P*katG*-SCFP3A kan (SU620)	Lagage et al.^34^	N/A
AB1157, Δ*flhD*, P_RNAI_-mKate2, mutL-mYpet, carrying pUA066-P*ahpC*-SCFP3A kan (SU948)	This study	N/A
AB1157, Δ*flhD*, P_RNAI_-mKate2 P*grxA*-SCFP3A kan (SU880)	This study	N/A
AB1157, Δ*flhD*, mKate2, MutL-mYPet, Δ*oxyR,* pUA P*grxA*-SCFP3A kan + pUC18 amp encodes YPet preceeded by 11 aa linker followed by a kan cassette flanked by frt sites. (SU882)	This study	N/A

**Chemicals, peptides, and recombinant proteins**		

M9 minimal salts 5x	Sigma	M9956
MEM amino acids	Gibco	11130-036
L-Proline	Biochemica	A3453,0100
Thiamine	Biochemica	A0955,0050
Pluronic F-127	Sigma	P2443-250G
30% W/W solution of H_2_O_2_	Sigma	H1009-100mL
Propidium iodide	Sigma	P4170

**Software and algorithms**		

MATLAB	Mathworks	Mathworks.com
Python	Spyder	anaconda.com
BACMMAN	Ollion et al.^64^	github.com/jeanollion/bacmman

**Deposited data**		

All raw data collected for this study along with the custom-built python code for analysis	This study	https://doi:10.5287/bodleian:MzeKjr4XN

### Resource availability

#### Lead contact

Additional information regarding methods and materials used for the study can be directed to the lead contact, Stephan Uphoff (stephan.uphoff@bioch.ox.ac.uk).

#### Materials availability

Plasmids used in this study were obtained from the *E. coli* promoter library of pSC101 plasmids.^49^ *E. coli* strains that were constructed for this study can be obtained from the lead contact upon request and completion of the Materials Transfer Agreement.

### Experimental model and subject details

Experiments were performed with bacterial strains derived from *E. coli* K12 AB1157. Details about genetic modifications and growth conditions are described below.

### Method details

#### Strains and plasmids

The strains constitutively expressed P_RNAI_-mKate2 fluorescent marker for cell segmentation analysis and the *flhD* gene was deleted to inhibit flagellar motility allowing growth in microfluidic chips. This strain also carried an endogenous MutL-mYPet fusion for detection of DNA mismatches.^58^ The strain SU802 with a Δ*oxyR*::kan deletion was described in.^34^ The strain 882 with a Δ*oxyR*::kan deletion also carried a pUC18 plasmid expressing YPet as a fluorescent marker to distinguish Δ*oxyR* cells from wild-type cells in the experiments with a mixture of the two strains.

The OxyR response reporter plasmids (P*grxA*-CFP, P*katG*-CFP and P*ahpC*-CFP) were derived from an *E. coli* promoter library of pSC101 plasmids.^49^ Each plasmid in the library contains the promoter region of a specific gene or operon in front of GFPmut2 fluorescent protein. To avoid overlap with the yellow fluorescence channel for imaging MutL-mYPet, we changed the GFPmut2 to the fast-maturing cyan fluorescent protein SCFP3A using Gibson Assembly (NEB). The promoter regions in the reporter plasmids were sequenced and plasmids were transformed into the strain SU178. Presence of the expected fluorescent protein signals were verified by taking microscopy snapshots. Strains were selected for and subsequently grown in LB, or LB agarose supplemented with 25 μg/mL kanamycin.

#### Media and growth conditions

Strain construction was performed in LB or LB supplemented with antibiotics at 37°C. 4 mL cultures were grown at 200 rpm in 10 mL culture tubes. Successfully constructed strains were stored in glycerol stocks at −80°C. For experiments, strains were streaked from these glycerol stocks on LB agarose plates with appropriate antibiotic selection. A single colony was picked and then grown overnight in M9 minimal media. This media was prepared with M9 salts (15 g/L KH_2_PO_4_, 64 g/L Na_2_HPO_4_, 2.5 g/L NaCl, and 5.0 g/L NH_4_Cl), 2 mM MgSO_4_, 0.1 mM CaCl_2_, 0.5 mg/mL thiamine, MEM amino acids, 0.1 mg/mL L-proline, and 0.2% glucose. The next day, overnight culture was diluted 1:50 and grown to OD600–0.3 in M9 minimal media. For loading cells in microfluidic chips, 0.85 mg/mL Pluronic F127 was added to the media to avoid cell aggregation. For experiments done under hydrogen peroxide treatment, the specific concentration of H_2_O_2_ was added to the growth media immediately before the start of the experiment.

#### Microfluidic chip preparation

Single-cell imaging was performed using the ‘mother machine’ microfluidic device as described in.^34^^,^^48^ The chip has a main channel for flow of media with dimensions 25 μm width and 100 μm height. This main channel is branching into perpendicular growth channels (here called ‘growth trenches’) of dimension 1.2 μm width and 1.2 μm height and 25 μm length. Where indicated, a different silicon wafer was used with larger trenches of 1.4 μm width and 1.4 μm height and 25 μm length. The chips were made of polydimethysiloxane (PDMS) polymer using a silicon wafer mold (Conscience). A 1:10 solution of polymerising agent and PDMS monomer were rigorously mixed and then poured onto the silicon wafer. This was placed in a vacuum chamber and pressurised to remove air bubbles. The device was then heated at 65°C in an oven for 2 h to polymerise. For each experiment, one chip was cut out using a scalpel, and holes for inlet and outlet were inserted using a 0.75 mm biopsy puncher. The device was cleaned using 100% ethanol and dried with nitrogen gas. The cleaning was repeated 3 times. The PDMS chip was bonded on a glass coverslip (thickness No 1.5). These coverslips were first cleaned by sonication with acetone for 20 min followed by isopropanol for 20 min, and then dried with nitrogen gas. The cleaned coverslip and PDMS chip were exposed to air plasma for 2 min and bonded at 95°C for 30 min.

#### Mother machine setup

1 mL of exponentially grown cells were spun down for 2 min at 6000 rpm. These cells were then resuspended in 100 μL of the supernatant and loaded in the microfluidic chips by pipetting through the inlet. For experiments with a lower and variable number of cells per trench, the cells were centrifuged and resuspended in 500 μL of supernatant before being loaded into the chip. The chip was then inserted into a custom-built centrifuge holder and spun at 5000 rpm for 10 min to aid the loading of cells into the growth trenches. 50 mL syringes were filled with M9 minimal media containing Pluronic F127 and H_2_O_2_ as indicated. The syringes were attached to silicon tubing (Tygon) and loaded onto syringe pumps (NewEra SyringePumpPro) to deliver media into chips at a constant flow rate of 2.5 mL per hour. Cells were initially grown without H_2_O_2_ for ∼3 h before switching the inlet media to a syringe containing H_2_O_2_ using a Y-junction attached to the inlet of the chip.

#### Live dead staining

1 mg/mL solution of propidium iodide was prepared from the stock. 1/1000 dilution was added in PBS buffer solution and flown into the mother machine 11.5 hours after treatment removal, images of which were captured in the RFP channel.

#### Cell fixation

Inert cells were prepared by chemical fixation. Cells were pelleted and resuspended in 4% formaldehyde, and then stored at room temperature for 30 minutes for inactivation before mixing with live WT cells for imaging.

#### Time-lapse microscopy

Time lapse imaging was performed using a Nikon Ti-E inverted fluorescence microscope equipped with 100x NA 1.40 immersion oil objective, motorized stage, sCMOS camera (Hamamatsu Flash 4), LED excitation source (Lumencor SpectraX), and operated with a perfect focus system. Exposure times were 100 ms for P_RNAI_-mKate2 (λ = 555 nm), 75 ms for CFP reporters (λ = 440 nm) and 300 ms for MutL-mYPet (λ = 508 nm) using 50% of maximal LED excitation intensities. The excitation and emission lights were separated using a triband dichroic and individual emission filters. The microscope chamber (Okolabs) was maintained at 37°C throughout the experiments. Images were captured every 3 min for the 3 emission channels. On average, 45 to 50 fields of view (FOV) were captured per experiment with each FOV containing 26 growth trenches. This yielded ∼1200 independent growth trenches each containing a single mother cell and its progeny. With 8 cells per growth trench, on average ∼10,000 cells were captured in total per time point. For cross correlation analysis experiments, we imaged P_RNAI_-mKate2 for cell segmentation at 50 ms and CFP channel at 50 ms exposure times every 45 seconds. Since this was a shorter time duration between cycles, we could capture around 18 to 20 FOV which yielded ∼500 cells per time point.

#### Imaging of microcolonies

Cells were streaked on LB plates with antibiotics (25 μg/mL kanamycin and 100 μg/mL ampicillin) and grown overnight at 37°C. A colony was picked and grown overnight in 4 mL LB at 37°C in a shaking culture. The next day, 2 μl spots of overnight culture were dropped on a LB agarose plate without letting the pipette tip touch the agar surface and grown for 2 hours at 37°C. Using a scalpel, the agarose was cut surrounding a spot (1 cm by 1 cm). 8 μl of 10 mM H_2_O_2_ solution in LB was dropped close to the spot and let to dry for ∼3 minutes. The agarose pad was then flipped onto a clean glass slide and then covered with a cap of a culture tube that was sealed with tape. Time lapse movies were performed on a Nikon Ti-E microscope equipped with a 100x NA 1.45 oil immersion objective, motorised stage, sCMOS camera (Photometrics Prime95B), LED excitation source (Lumencor SpectraX) and perfect focus system. Exposure times were 100 ms for P_RNAI_-mKate2 (λ = 555 nm), 75 ms for CFP reporters (λ = 440 nm) and 300ms for MutL-mYPet (λ = 508 nm) using 50% of maximal LED excitation intensities. The microscope chamber (Okolabs) was maintained at 37°C throughout the experiments. Images were captured every 3 minutes for brightfield and the 3 fluorescence channels. On average, 10 to 20 fields of view (FOV) were recorded per experiment with each FOV capturing a section of the microcolony.

### Quantification and statistical analysis

#### Statistical analysis

Number of biological replicates and number of cells per analysis are given in the figure legends. Statistical analysis was performed on data sets using the stats library in Python. ^∗^p ≤ 0.05, ^∗∗^p ≤ 0.01, ^∗∗∗^p ≤ 0.001 and ^∗∗∗∗^p ≤ 0.0001 were considered as significant. The type of statistical analysis is stated in the figure caption for each plot. Linear regression fits were performed using the LinearRegression module of the sklearn.linear_model library in Python. Exponential fits were performed in MATLAB using Curve Fitter app.

#### Mother machine data processing and analysis

Time lapse microscopy data were saved as.nd2 files and visualized in Fiji. The data were processed using the BACMMAN plugin in Fiji as described in^64^ and further analyzed using custom Python scripts [Data S1G]. Graphs were generated using MATLAB. Images were first pre-processed by BACMMAN using the P_RNAI_-mKate2 fluorescence channel to stack all individual growth trenches and correct for experimental drift in x-y coordinates and image rotation. The outlines of cells in the growth trenches were then jointly segmented and tracked over time based on the P_RNAI_-mKate2 fluorescence signal. The traces were visually inspected and manually corrected for errors in segmentation or lineage tracing using the BACMMAN software. The CFP fluorescence of the different reporter plasmids was extracted by overlaying the cell masks from the P_RNAI_-mKate2 channel onto the CFP channel and computing the mean intensity over the cell area. BACMMAN software was also used to detect foci of the MutL-mYPet reporter for DNA mismatches within the cell masks. BACMMAN generated output in 4 excel files containing cell growth characteristic, P_RNAI_-mKate2 intensity data, CFP intensity data, and MutL-mYPet foci detections. These files were then further analyzed using a custom python pipeline as described in the following.

#### Cell parameter calculations

##### Cell length (L)

The cell length was computed from the maximum distance between the points on the cell masks.

##### Cell area (A)

The cell area was computed from the total area covered by the cell mask.

##### Estimated cell surface area to volume ratio

This ratio was empirically calculated as: $\rho =\frac{24L\;\left(L^{2}+A\right)}{A\left({3L}^{2}-A\right)}$.

A cell is considered as a cylinder capped with 2 hemispheres with the total length as L and radius as r.

$$\frac{SA}{V}=\rho =\frac{2\pi r\left(L-2r\right)+4\pi r^{2}}{\pi r^{2}\left(L-2r\right)+\frac{4\pi r^{3}}{3}}$$

Simplifying the above equation and approximating A=2rL. Here A is the cell area output from BACMMAN.

$$\rho =\frac{24L\;\left(L^{2}+A\right)}{A\left({3L}^{2}-A\right)}$$

##### Elongation rate

The instantaneous elongation rate was calculated based on the log-difference in cell length between consecutive frames as $\frac{\log\left(L_{t}\right)-\log\left(L_{t-\Delta t}\right)}{\Delta t}\text{.}$ For calculating the elongation rates of cells at different positions in the growth trench (e.g. Figure 1C), cells were tracked according to their initial position until the number of barrier cells decreased by 2.

##### Generation time

The time difference between two consecutive cell divisions.

##### Division rate

The inverse of the cell generation time.

##### Reporter fluorescence intensities

The intensity values were averaged over the area of each cell.

##### Length growth rate

This was calculated as the coefficient of a linear fit (β) to the logarithm of cell length over each cell cycle. L = L_0_e^βt^, where L_0_ is the length at birth. Since, it is calculated over the whole lineage duration, we cannot capture the instantaneous changes in elongation rate.

##### Fate of a cell

A mother cell was defined dead if the length growth rate was less than 0.012 min^−1^ for >10 hours after stress removal.

##### Number of barrier cells

The number of cells that are located between the open end of a trench and the cell being analyzed.

##### Cumulative barrier length

The sum of lengths of barrier cells.

##### Cumulative barrier area

The total area covered by the barrier cells.

##### Cumulative barrier surface area to volume

The total estimated surface area to volume ratio of all barrier cells.

##### Lineage annotation

Identity of cells was obtained from BACMMAN to define their lineage relations as sisters, cousins, or non-related.

#### Stress response parameter calculations

##### Response peak intensity

The oxidative stress response reporters showed an initial peak in intensity and then reached a lower steady-state level. The initial peak intensities for each mother cell were detected using the PeakUtils module in Python with a normalised threshold of 0.15 and minimum distance between peaks of 2 frames. This absolute response peak value was subtracted by the mean reporter intensity without H_2_O_2_ treatment.

##### Response induction time

The time at which a mother cell crosses an intensity threshold of 20% of the steady state intensity value (i.e. 1480 a.u. for P*grxA*-CFP) after the start of H_2_O_2_ treatment.

##### Response peak time

The time at which a mother cell reached its response peak intensity after the start of H_2_O_2_ treatment.

##### Coefficient of variation

The CV values were calculated as the mean divided by the standard deviation for the response induction time, peak time, and peak intensity. CVs of steady state intensities were calculated for mother cells or all cells from 90 minutes post treatment until 6 hours post treatment after subtracting the mean intensity for each cell before H_2_O_2_ treatment.

#### Cross-correlation analysis

The temporal cross-correlation between the reporter intensity traces of mother cells and the number of barrier cells per trench was computed using the statsmodel library in Python. Correlation values from individual growth trenches were then averaged over all observed growth trenches for different lag times. The minimum of the cross-correlation curve was obtained using the argrelextrema function in Python to obtain the lag time of the reporter intensity in response to changes in the number of barrier cells.

#### Calibration of H_2_O_2_ concentration inside growth trenches

We estimated the local H_2_O_2_ concentration in the growth trenches from the P*grxA*-CFP reporter intensities of cells located at different positions. We calibrated the analysis using the reporter intensities of the cells located at the open end of the growth trenches that were exposed to defined H_2_O_2_ concentrations (12.5 μM, 25 μM, 37.5 μM, 50 μM, 62.5 μM, 75 μM and 100 μM). The fluorescence intensities at steady-state (1–3 hours after start of treatment) showed a linear relation with the external H_2_O_2_ concentration. This was used to obtain the conversion factor for computing the H_2_O_2_ concentration from the reporter intensity of cells at any position in the trenches.

#### Machine learning

We trained a random forest regressor model to predict the initial peak intensity of the P*grxA*-CFP reporter for each mother cell according to the schematic in Figure 2A. A separate random forest classifier model was trained to predict the external H_2_O_2_ concentration. A list of features (shown below) was calculated from the output data provided by BACMMAN. Data from multiple experiments (as indicated in figure captions) were combined into one dataset for model training and testing. Feature values for each mother cell and its environment were stored in separate CSV files which served as input to the Python-based Machine learning model. The following Python libraries/packages were used: pandas; numpy; peakutils; matplotlib; stats, from scipy; preprocessing, utils, shuffle, metrics, model_selection, ensemble from sklearn, ststamodel.api.

126 features were extracted from each mother cell and labeled with the output value i.e. the P*grxA*-CFP peak intensity for each cell. The data was then split 80:20 into training and test data using train_test_split module from sklearn. The training and test data were then individually shuffled using the shuffle library in sklearn.

Utilising the supervised learning method, a random forest model is an ensemble technique that can predict the output numerical value or classification class, for regressor and classifier respectively, based on the combined outputs generated by multiple decision trees that each process a random subset of the data with a random subset of the available features.^65^

For the regression model, the decision trees compute numerical values at each tree node. The feature values were extracted from a subset of training data at each decision node that were randomly drawn from the feature list. Prediction was then performed for multiple trees and mean squared error was calculated for every predictor and minimized by bootstrapping. The final output numerical value for the P*grxA*-CFP peak intensity per cell is the average of these predictions.

The classification model generates probabilistic predictions of labelled outputs of the given dataset, whose decision is based on bootstrapping results from a collection of randomized trees. Here, the different H_2_O_2_ treatment concentrations were considered as separate classes for each mother cell. The decisions trees making up the forest were trained with the input data as an independent classifier. These independent trees are formed using random subsets from training datasets and the randomized feature set that was extracted. The label that is selected by the majority of the trees is output as the class.

We used 100 trees and forest depth of 3000 for bootstrap samples in both of the above-mentioned algorithms.

After the models had been trained, the test data were used to evaluate the model accuracy based on the normalized difference between the predicted and observed P*grxA*-CFP peak intensity per cell, and averaged over all cells in the test dataset. We report the model accuracy accordingly:

$$A=1-\frac{1}{n}.\sum_{s=1}^{s=n}|\frac{{pred}_{s}-\exp_{s}}{\exp_{s}}|$$

The accuracy of the classification model was evaluated using a confusion matrix to quantify the frequency of true positive and true negative predictions. To assess the relative importance of the different feature types for the predictive power of the model, we used the mean decrease impurity test in the scikit-learn package in Python.^65^

#### Machine learning features

${GrxA}_{peak}={\left\{{GrxA|}_{t_{p}<t<t_{q}}\right\}}_{peak}-\frac{\sum_{t=-treatment}^{t=0}GrxA}{treatment};t_{p}=0,t_{q}=60\;minutes$

1. Kurtosis = $Y_{kurt}=\left(t_{2}-t_{1}\right)*\frac{\sum_{t=t_{1}}^{t=t_{2}}\left(Y_{t}-\frac{\sum_{t=t_{1}}^{t=t_{2}}Y_{t}}{t_{2}-t_{1}}\right)}{{\left(\sum_{t=t_{1}}^{t=t_{2}}\left({\left(Y_{t}-\frac{\sum_{t=t_{1}}^{t=t_{2}}Y_{t}}{t_{2}-t_{1}}\right)}^{2}\right)\right)}^{2}}$
2. Skewness = $Y_{skew}=\left(t_{2}-t_{1}\right)*\frac{\frac{\sum_{t=t_{1}}^{t=t_{2}}Y_{t}}{t_{2}-t_{1}}-Median\left(Y_{t}\forall t_{1}<t<t_{2}\right)}{{\left(\frac{\sum_{t=t_{1}}^{t=t_{2}}{\left(Y_{t}-\frac{\sum_{t=t_{1}}^{t=t_{2}}Y_{t}}{t_{2}-t_{1}}\right)}^{2}}{t_{2}-t_{1}}\right)}^{0.5}}$
3. Median = $Y_{median}=Median\left(Y_{t}\forall t_{1}<t<\;t_{2}\right)$
4. Mean or average = $Y_{mean}=\frac{\sum_{t=t_{1}}^{t=t_{2}}Y_{t}}{t_{2}-t_{1}}$
5. Minimum = $Y_{\min}=Minima\left(Y_{t}\forall t_{1}<t<\;t_{2}\right)$
6. Maximum = $Y_{\max}=Maxima\left(Y_{t}\forall t_{1}<t<\;t_{2}\right)$
7. Range of magnitude = $Y_{range}=Y_{\max}-Y_{\min}$

All the above features were calculated for ‘mother cells’ where $Y_{t}$ = :

1. Cell length at time point t = $l_{t}$
2. Cell area at time point t = $A_{t}$
3. Instantaneous cell elongation rate at time point t = $\beta_{t}=\frac{l_{t}-l_{t-1}}{1}$
4. Instantaneous cell area increase rate at time point t = $\rho_{t}=\frac{A_{t}-A_{t-1}}{1}$
5. Estimated cell surface area to volume ratio at time point t = $\alpha_{t}=\frac{l_{t}}{A_{t}-\left(\frac{2{A_{t}}^{2}}{3{l_{t}}^{2}}\right)}$
6. Number of barrier cells at time point t = $B_{t}={Maxima(Idx}_{cell,t})-1$
7. Total length of barrier cells = $l_{t,barrier}={\left(\sum_{i=0}^{i=B_{t}}l_{i}\right)}_{t}$
8. Total area of barrier cells = $A_{t,barrier}={\left(\sum_{i=0}^{i=B_{t}}A_{i}\right)}_{t}$
9. Cumulative 3D surface area to volume ratio of barrier cells = $\alpha_{t,barrier}={\left(\sum_{i=0}^{i=B_{t}}\frac{l_{i}}{A_{i}-\left(\frac{2{A_{i}}^{2}}{3{l_{i}}^{2}}\right)}\right)}_{t}$

For accounting for different conditions, the following time point series were analyzed.1

1. Untreated: $t_{2}=treatment,t_{1}=treatment-135\;minutes$
2. Hydrogen peroxide treated: $t_{2}=treatment+135\;minutes,t_{1}=treatment$

Total number of input features = 7^∗^9^∗^2 = 126 + 1 output variable = 127 features.

#### Microcolony image analysis

Segmentation of cells growing in microcolonies was performed based on the P_RNAI_-mKate2 fluorescence signal and using the MicrobeTracker tool in MATLAB^66^ followed by manual correction of the segmentation masks. These outlines were then applied to the CFP channel and a MATLAB script was used to quantify the average intensity per cell area.

#### MutL-mYPet foci detection

MutL-mYPet foci detection was carried out by BACMMAN. Images were band-pass filtered between within 1–400 pixels. Next, the image was Gaussian smoothed with a scale of 2 pixels and the spot segmentation was performed using Seed Laplacian threshold of 0.8, propagation threshold of 1.5 and seed threshold of 1.7. Foci that were detected in multiple consecutive frames within the same cell were counted only once in the first frame. The mismatch rate was calculated by dividing the total number of foci by the total number of cells per frame.

## Data Availability

• All data reported in this paper will be shared by the lead contact upon request. • All raw data collected for this study along with the custom-built python code used for analysis have been deposited and are publicly available on the Oxford Research Archive: https://doi:10.5287/bodleian:MzeKjr4XN. • Any further information required for reanalysing the data reported in this paper is available upon request directed to the lead contact.

## Supplemental information

Document S1. Figures S1–S6

Data S1. Excel files with machine-learning parameters and results and BACMMAN configuration file Data S1A. Machine learning regressor model feature importance list, related to Figure 2. The description for all features is in the material and methods section. Data S1B. Machine learning regressor model: Predicted and experimental P*grxA* peak values for test data output by the regressor model, related to Figure 2. Data S1C. Machine learning classifier model: Predicted and experimental H2O2 concentration values for test data output by the classification model, related to Figure S2A. Data S1D. Machine learning classifier model feature importance list, related to Figure S2A. The description for all features is in the material and methods section. Data S1E. Machine learning regressor model trained only on barrier cell features: Predicted and experimental P*grxA* peak values for test data output by the regressor model trained only on barrier cell features, related to Figure S2B. Data S1F. Machine learning regressor model feature importance list for model trained only on barrier cell features. related to Figure S2B. The description for all features is in the material and methods section. Data S1G. BACMMAN configuration file: Contains parameters used for cell segmentation and lineage tracing with BACMMAN software, related to materials and methods.

Document S2. Article plus supplemental information
