# Supplementary material for: Phenotypic heterogeneity in the bacterial oxidative stress response is driven by cell-cell interactions
Source: Cell Rep. 2023 Feb 26;42(3):112168. doi: 10.1016/j.celrep.2023.112168 (PMC10935545; doi:10.1016/j.celrep.2023.112168)
Supplement: Document S1. Figures S1–S6 [file mmc1.pdf]

Cell Reports, Volume *42*

Supplemental information

Phenotypic heterogeneity in the bacterial oxidative stress response is driven by cell-cell interactions

Divya Choudhary, Valentine Lagage, Kevin R. Foster, and Stephan Uphoff

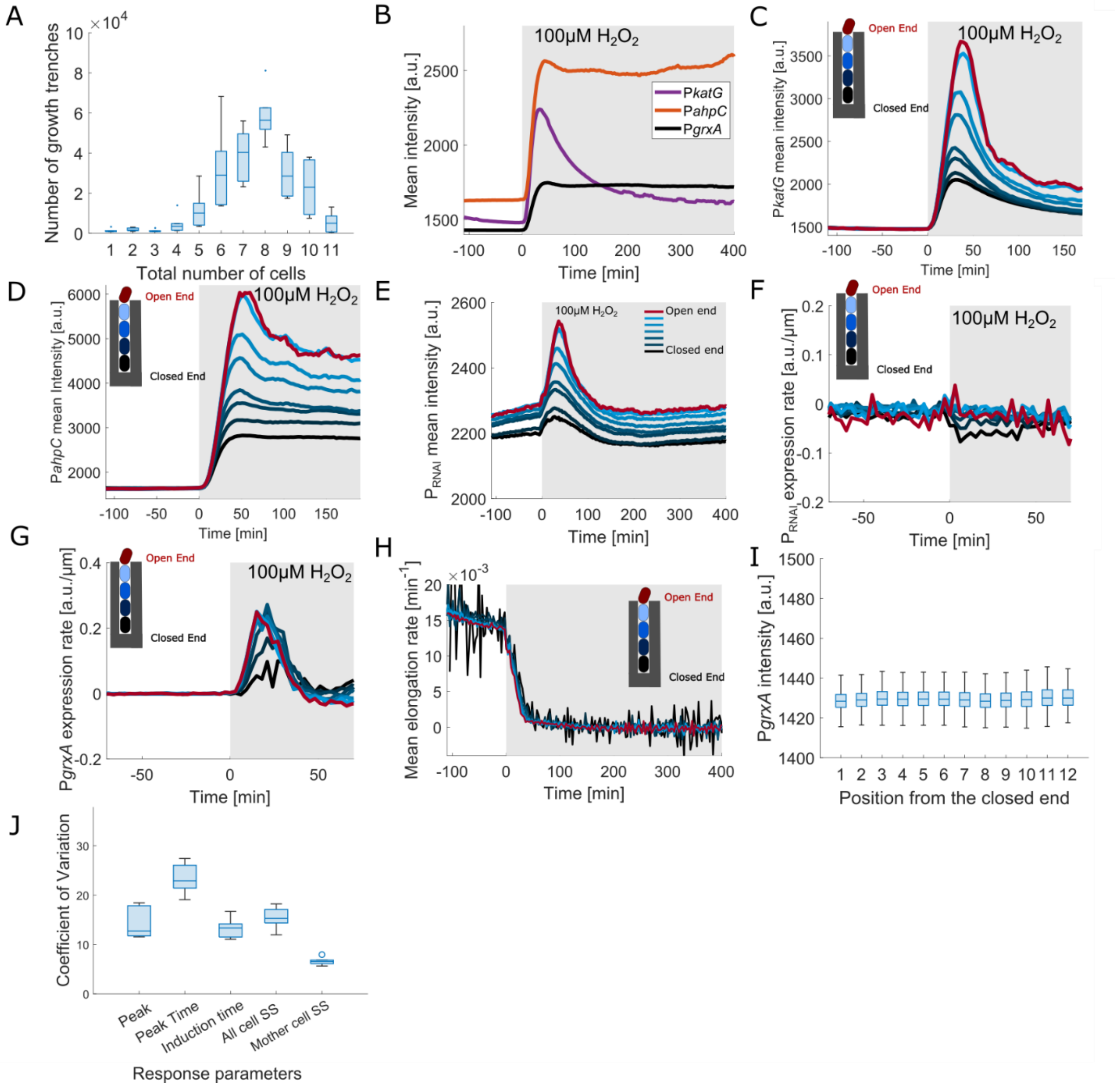

Figure S1: **Oxidative stress response characterised in microfluidic experiment:** Related to figure 1. (A) Distribution of number of cells per growth trench at the time of 100 µM H_2O_2 treatment (3 experimental repeats). (B) Mean CFP intensity for mother cells under 100 µM H_2O_2 treatment added at time 0 min (shaded area) for the transcriptional reporters of P*katG* and P*ahpC* and P*grxA* ($\geq$ 2 experimental repeats each). (C) P*katG*-CFP intensities with continuous 100 µM H_2O_2 treatment added at time 0 min (shaded area) averaged across cells at specific positions in the growth trench (2 experimental repeats, black line: mother cells at closed end; red line: cells at open end). (D) P*ahpC*-CFP plot similar to panel C (2 experimental repeats). (E) P_{RNAI}-mKate2 plot similar to panel C (3 experimental repeats). (F) P_{RNAI}-mKate2 expression rate (promoter activity) with continuous 100 µM H_2O_2 treatment added at time 0 min (shaded area) averaged across cells at specific positions in the growth trench (3 experimental repeats, black line: mother cells at closed end; red line: cells at open end). (G) P*grxA*-CFP expression rate (promoter activity) similar to panel F (3 experimental repeats). (H) Mean elongation rate for Δ*oxyR* cells at different positions in the growth trench with 100 µM H_2O_2 treatment (Movie S2) (shaded area; black line: mother cells at closed end; red line: cells at open end) (3 experimental repeats). (I) P*grxA*-CFP intensities for cells at different positions in the growth trench before treatment (3 experimental repeats, box plots with median 25th and 75th percentile). (J) Variation of the oxidative stress response across mother cells with 100 µM H_2O_2 treatment. Coefficient of Variation (standard deviation/mean) for the peak amplitude (Peak), the time to reach the PgrxA-CFP peak intensity (Peak Time), the response induction time (time until P*grxA*-CFP > 1480 a.u.) and P*grxA*-CFP intensity from 2 hours post treatment (SS: steady-state) for all cells in growth trenches and for all mother cells (3 experimental repeats, box plots with median 25th and 75th percentile).

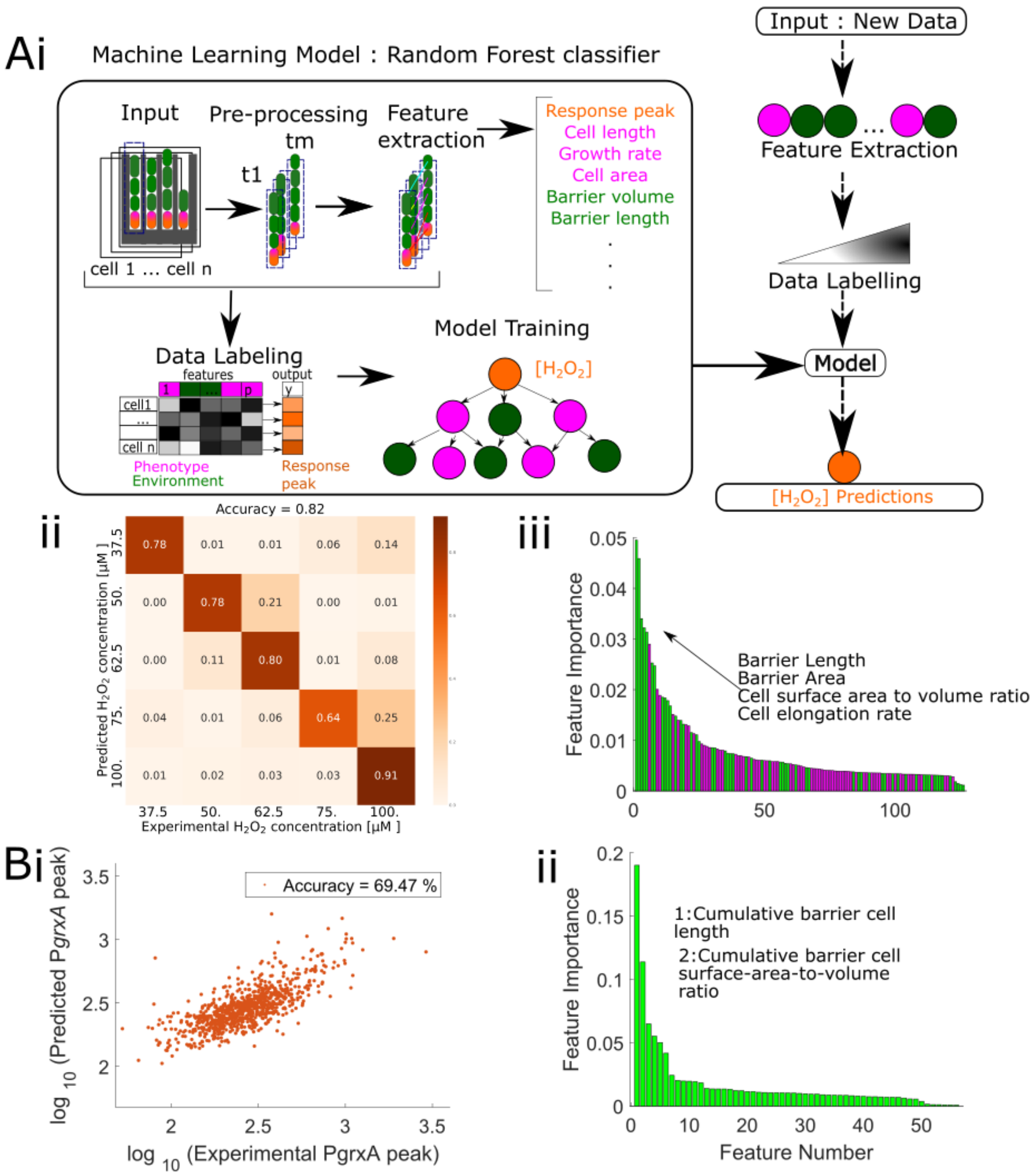

Figure S2: **Machine learning models to predict oxidative stress response heterogeneity and H_2O_2 treatment concentrations:** Related to figure 2. (A) Machine learning classifier model predicts external H_2O_2 concentration(orange). (i) It uses features that describe the phenotypic characteristics of the mother cell (magenta) and the other cells in the local environment of each trench (barrier cells, green). (ii) Confusion matrix of predicted [H_2O_2] against the experimental [H_2O_2] for ~900 mother cells (unseen by training data) (data shown in Data S1C). (iii) Feature importance plot shows the relative contribution of the 126 input features to the predictive power of the model. Mother cell features shown in magenta and local environment features (relating to the barrier cells) in green. The features whose mathematical derivatives are in the top 10 most important features are highlighted. Whereas the prediction of P*grxA*-CFP peak intensities relied on only a few important features relating to the barrier cells, prediction of [H_2O_2] uses a broader range of different features relating to the mother cell and barrier cells (feature names in Data S1D). (B) Machine learning model trained only on barrier cells predicts mother cell response heterogeneity: A random forest machine learning model predicts P*grxA*-CFP peak intensities of ~850 mother cells (orange). It uses features that describe the other cells in the local environment of each trench (barrier cells, green), and no features for the mother cells themselves. (i) P*grxA*-CFP peak predicted by that model plotted against the experimentally measured P*grxA*-CFP peak (each dot represents one mother cell) (data in Data S1E). (ii) Feature importance plot shows the relative contribution of the 54 input features to the predictive power of the model. The features of the two most important environmental characteristics accounting for ~73.5% importance are highlighted (feature names in Data S1F).

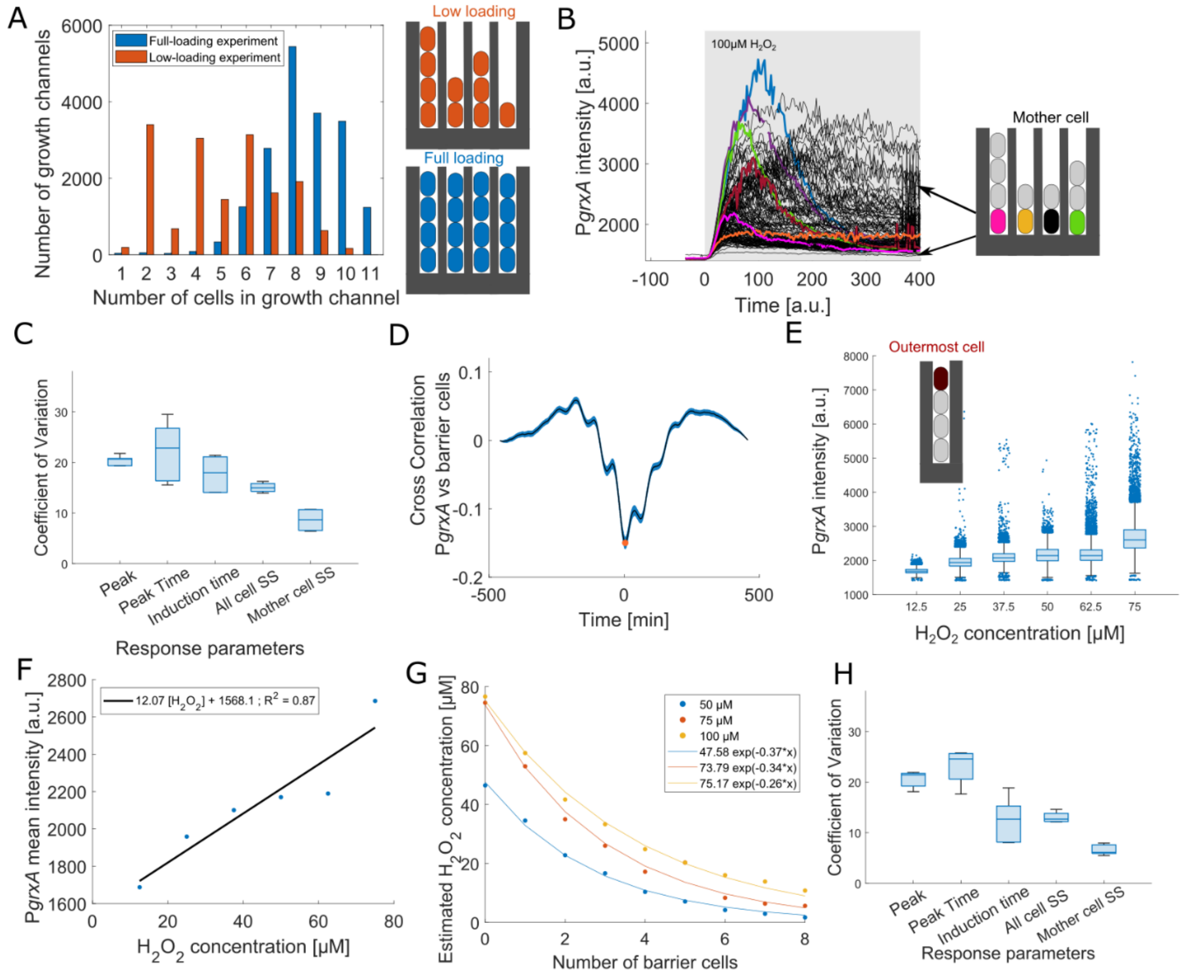

Figure S3: Variation in the number of barrier cells per growth trench increases response heterogeneity across mother cells: Related to figure 3. (A) Distribution of number of cells per trench at the beginning of treatment for experiments with partially filled trenches (low-loading, orange) versus completely filled trenches (full-loading, blue) (3 experimental repeats each). (B) P*grxA*-CFP intensities of individual mother cells (6 example cells highlighted in colour) over time treated with 100 µM H_2O_2 (added at time 0 min, shaded area) (~300 traces). (C) Variation of the oxidative stress response across mother cells for low-loading experiment after 100 µM H_2O_2 treatment. Coefficient of Variation (standard deviation/mean) for the peak amplitude (Peak), the time to reach the PgrxA-CFP peak intensity (Peak Time), the response induction time (time until P*grxA*-CFP > 1480 a.u.) and P*grxA*-CFP intensity from 2 hours post treatment (SS i.e. steady state) for all cells in growth trenches and for all mother cells (3 experimental repeats, box plots with median 25th and 75th percentile). (D) Mean temporal cross correlation for P*grxA*-CFP of mother cells against the number of barrier cells per trench shown in black (the blue shadow shows the SEM across all traces, example time traces shown in Figure 3C), when mean P*grxA*-CFP intensity has reached steady-state from 2 hours after start of 100 µM H_2O_2 treatment until end of experiment (~11 hours) (~950 cells, 2 experimental repeats). Measurements were performed with 45 second time interval between frames. (E) P*grxA*-CFP intensities for these outermost cells 2 hours after the start of treatment for different H_2O_2 concentrations. (~107500 data points with 17921 ± 1960 data-points for each concentration, box plots with median 25th and 75th percentile) (F) A linear regression fit of the mean values in panel E gave the calibration equation for intensity changes for different H_2O_2 concentrations as $I = 12.07 \cdot [H_2O_2] + 1568.1$ with $R^2=0.87$. (G) Estimated concentration along the growth trench with mean intensities for varying number of barrier cells based on the PgrxA-CFP intensity according to the calibration curve from panel F. Single exponential fits are shown. (H) Variation of the oxidative stress response across mother cells in 1.4 µm wide growth trenches after 100 µM H_2O_2 treatment. Coefficient of Variation (standard deviation/mean) for the peak amplitude (Peak), the time to reach the P*grxA*-CFP peak intensity (Peak Time), the response induction time (time until P*grxA*-CFP > 1480 a.u.) and P*grxA*-CFP intensity from 2 hours post treatment (SS: steady-state) for all cells in growth trenches and for all mother cells (3 experimental repeats, box plots with median 25th and 75th percentile).

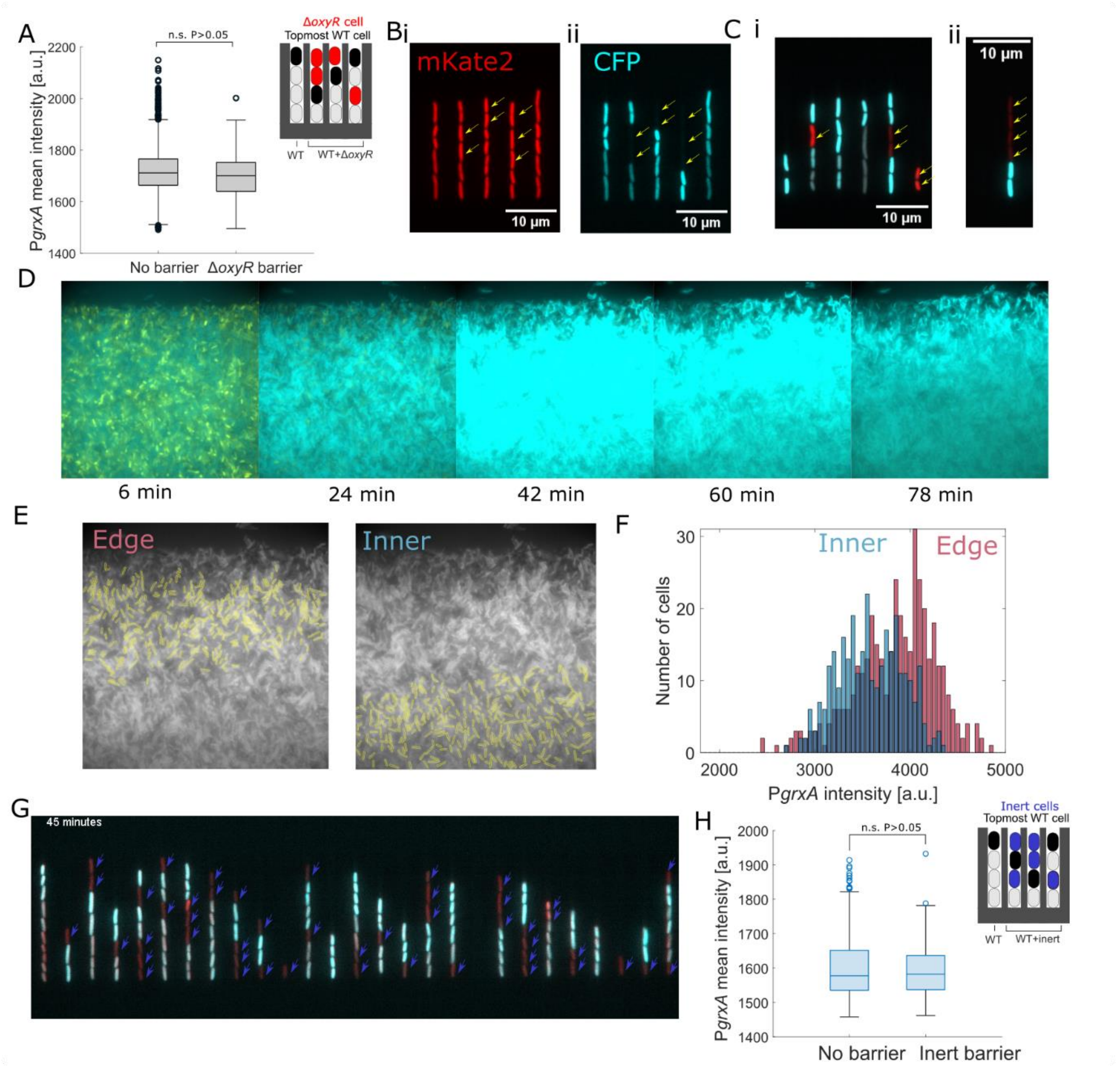

Figure S4: **Δ*oxyR* or metabolically inactive cells do not provide cross protection against H_2O_2 stress:** Related to figure 4. (A) P*grxA*-CFP intensity ~20 min post 100 µM H_2O_2 treatment for top-most WT cells (shown as black in the schematic) in trenches that have either no barrier or barrier of Δ*oxyR* cells. (3 experimental repeats, box plots with median 25th and 75th percentile). (B) Snapshot of P*grxA*-CFP (cyan) and mKate2 cell marker (red) intensity for trenches with a mix of WT and Δ*oxyR* strain under 100 µM H_2O_2 treatment (Δ*oxyR* cells marked with arrow). (C) Merged snapshot P*grxA*-CFP (cyan) and mKate2 cell marker (red) intensity for trenches with a mix of WT and Δ*oxyR* strain under 100 µM H_2O_2 treatment (Δ*oxyR* cells marked with arrow). (D) P*grxA*-CFP snapshots of a microcolony of WT mixed with Δ*oxyR* cells (yellow) under 10 mM H_2O_2 treatment. (E) Segmented WT cells on the edge or interior of the microcolony. (F) Histograms of P*grxA*-CFP intensity for WT cells at the edge or interior of a microcolony after 30 min of 10 mM H_2O_2 treatment. (G) Snapshot of P*grxA*-CFP (cyan) and mKate2 cell marker (red) intensity for trenches with a mix of live WT cells and inert (chemically fixed) cells under 100 µM H_2O_2 treatment (inert cells marked with arrow). (H) P*grxA*-CFP intensity ~20 min post 100 µM H_2O_2 treatment for top-most WT cells (shown as black in the schematic) in trenches that have either no barrier or barrier of inert cells. (4 experimental repeats, box plots with median 25th and 75th percentile)

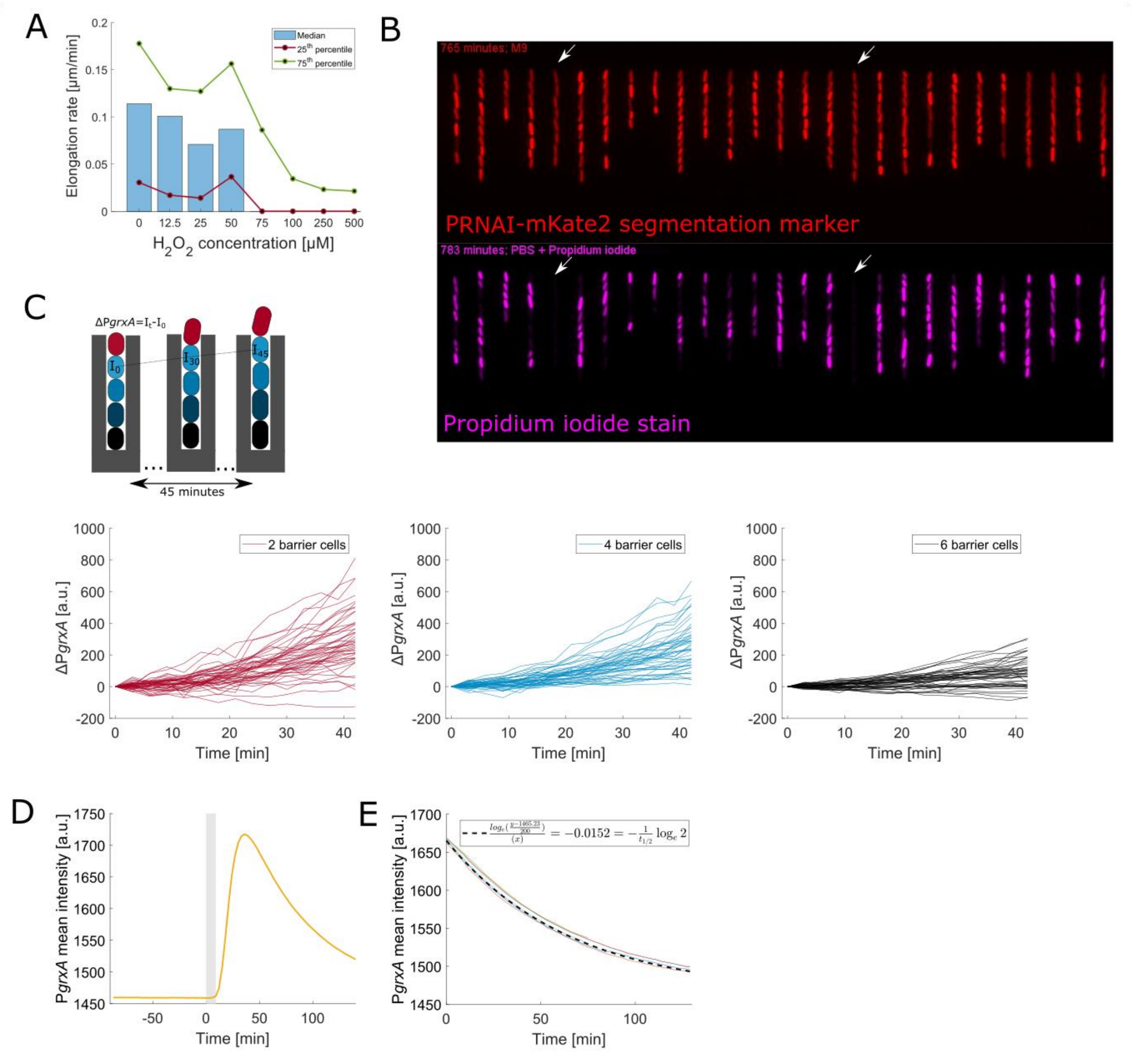

Figure S5: **Cell survival and effect of cell movement on P*grxA*-CFP expression in a H_2O_2 gradient:** Related to figure 5-6. (A) Elongation rate for outermost cells in the trenches traced from time of treatment until 30 minutes post treatment for different H_2O_2 concentrations. (B) (top) Snapshot of *E. coli* cells with segmentation marker P_{RNAI}-mKate2 in growth trenches 700 minutes (~11.5 hours) post removal of 500 µM H_2O_2 treatment. The 2 growth trenches marked with white arrows regrew after treatment removal as seen in Movie S6. (bottom) Snapshot of *E. coli* cells stained with propidium iodide as an indicator of cell death. (C) Increase in P*grxA*-CFP intensity for single cells (Δ*PgrxA*) over time when the response has reached steady-state (from 2 hours after start of 100 µM H_2O_2 treatment until end of experiment ~11 hours). Each curve represents a single cell moving towards the trench opening from a different starting position. (red line: cells with 2 barriers; blue line: cells with 4 barriers; black line: cells with 6 barriers; 45 traces shown for each case). (D) Mean P*grxA*-CFP intensity for 100 µM H_2O_2 treatment from 0 to 9 minutes (shaded). (E) Exponential fit to the decay of intensity after treatment removal of data shown in panel D, $t_{1/2}$ = 45.6 min as expected for the generation time of ~45 min (thin lines show curve of different dilution traces and bold dashed line indicates the fit, 6 repeats). This shows that sCFP3 is stable and diluted by cell growth.

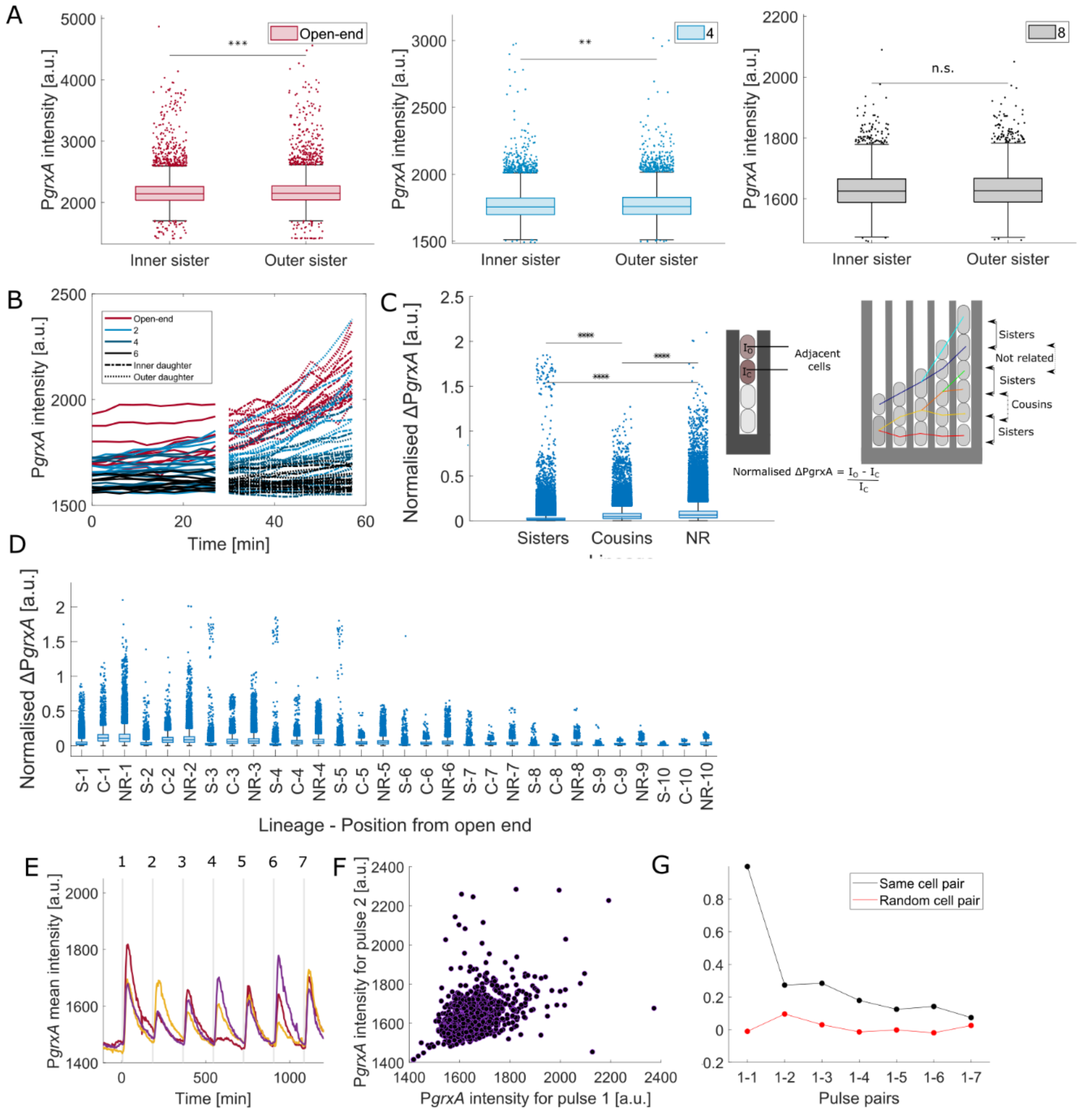

Figure S6: Cell memory in response to H_2O_2 treatment: Related to figure 6. (A) P*grxA*-CFP intensity at the time of division for sister cells at steady state with 100 μM H_2O_2 treatment. (Outer sister: closer to the open end). Pairs with outer sister having 0 (maroon), 4 (blue) and 8 (black) barrier cells are displayed (~ 34000 sister cell pairs, 3 experimental repeats, box plots with median 25th and 75th percentile; *** p<0.001, ** p<0.01 and n.s. for p>0.05). (B) Single-cell traces for the data shown in Figure 6F. P*grxA*-CFP intensity traces for sister cells (outer: dashed, inner: dash-dotted), and their progenitor cells (time of division ± 27 minutes). Colour code based on number of barrier cells at the time of division (10 mother-daughter pairs for each case plotted). (C) Normalised P*grxA*-CFP intensity difference for adjacent cell pairs related as sister (S), cousins (C), or not related (NR) under 100 μM H_2O_2 (box plots with median 25th and 75th percentile, **** p<0.0001, 3 experimental repeats). Cells become more correlated with closer lineage relation. (D) Normalised P*grxA*-CFP intensity differences for adjacent cells at different positions from the open end and separated by lineage identity (e.g. S-1 shows the intensity difference between sisters at position 1 from the open end etc; 3 experimental repeats, box plots with median 25th and 75th percentile). In general, unrelated cells have higher intensity differences than cousins or sisters, and intensity differences decrease with increasing number of barrier cells. (E) Testing for response memory with repeated short pulses of H_2O_2 treatment. Example P*grxA*-CFP traces of 3 mother cells experiencing 9 minutes of 100 μM H_2O_2 treatment every 3 hours (treatment times indicated in grey). (F) Scatter plot of P*grxA*-CFP intensity values of mother cells 60 minutes post treatment for the first and second pulse of treatment, showing ~25% correlation indicative of a low level of response memory between successive pulses (~800 cells, 2 experimental

repeats). (G) Correlation R values for P*grxA*-CFP intensity values for pairs of pulses of the same mother cell or for pulses of randomly paired cells as negative control (~800 cells, 2 experimental repeats). Intensities were measured at 60 minutes after each treatment pulse. Response memory decays over time from ~25% correlation between successive pulses (pulse pairs 1-2) close to the random level for pulse pairs 1-7. Residual long-lived correlation may reflect permanent intensity differences across cells e.g. due to position in the microscopy field of view.
